# The effectiveness of psychological interventions delivered by health professionals for adult cannabis use in community settings: a systematic review and meta-analysis

**DOI:** 10.3389/fpsyt.2026.1792228

**Published:** 2026-03-20

**Authors:** Sabrina Giguère, Stéphane Potvin, Mélissa Beaudoin, Xavier Wurtele Boudreau, Charles-Édouard Giguère, Alexandre Dumais

**Affiliations:** 1 Department of Psychiatry and Addictology, University of Montreal, Montreal, QC, Canada; 2 Research Center of the University Institute in Mental Health of Montreal, Montreal, QC, Canada; 3 Institut National de Psychiatrie Légale Philippe-Pinel, Montreal, QC, Canada

**Keywords:** cannabis-related problems, meta-analysis, psychological interventions, severity of cannabis dependence, systematic review

## Abstract

**Introduction:**

Cannabis is one of the most widely used psychoactive substances and is associated with negative consequences affecting physical and mental health, cognition, quality of life, functional outcomes, employment, and interpersonal relationships. The impact of cannabis use varies between individuals and is not necessarily related to consumption quantity or frequency. This variability underscores the importance of outcome measures beyond consumption patterns to capture real-world functional impact. Reducing the health and social consequences of substance use remains a core objective of many national drug strategies. Effective treatments for cannabis use are essential, as no pharmacological therapy is approved; psychosocial interventions remain the primary approach. This systematic review and meta-analysis aimed to summarize the evidence on psychological interventions delivered by health professionals in a community setting for adults with problematic cannabis use, focusing on outcomes related to cannabis-related problems and severity of dependence.

**Methods:**

A systematic search was conducted following the PRISMA guidelines. Articles were included if they quantitatively examined the efficacy of psychosocial interventions of at least two sessions, delivered by health professionals in community settings, among adult cannabis users. Meta-analyses were performed using random-effects models. Heterogeneity was assessed with I², and publication bias with Egger’s test. The risk of bias was assessed using the RoB 2 tool for randomized trials.

**Results:**

A total of fifteen studies were included, and the risk of bias was ranging from “some concerns” to “high”. Regarding cannabis-related problems and the severity of cannabis dependence, cognitive-behavioral therapy (CBT) was the only intervention associated with a small, temporary effect relative to active control groups (SMD=-0.23 to -0.36). Compared with inactive controls, CBT combined with motivational approaches showed small post-intervention benefits on cannabis-related problems (SMD=-0.39) and moderate to large effects on cannabis dependence (SMD=-0.69 to -0.88). However, most comparisons were non-significant, and most significant findings derived from a single study.

**Conclusion:**

Evidence supporting the effectiveness of psychological interventions for problematic cannabis use remains limited. Findings should be interpreted with caution, highlight the need for further high-quality research focusing on clinically meaningful outcomes beyond consumption patterns, as well as on the development and rigorous evaluation of innovative interventions targeting cannabis-related harms.

## 1 Introduction

Cannabis is one of the most psychoactive substances used worldwide. In 2022, approximately 228 million people reported using cannabis in the past year, representing about 4.4% of the global population aged 15 to 64 years (1). Globally, the number of individuals who have used cannabis in the past year has increased by nearly 28% between 2012 and 2022 (1). Among cannabis users, around 20% will develop cannabis use disorder (CUD) during their lifetime (2, 3). Furthermore, the rising THC potency observed over the past decade has been linked to an escalation in consumption patterns, a more rapid emergence of early CUD symptoms, and an elevated risk of problematic cannabis use (4–7). Independent of CUD, cannabis use is associated with various problems, including physical health issues (e.g., pneumonia, myocardial infarction), cognitive impairments (e.g., decision-making, processing speed, attention), and heightened risk of premature death (8–11). Cannabis use is often associated with a decrease in goal-directed motivation (amotivational syndrome), loss of interest in activities, and reduced social interactions, all of which contribute to a lower quality of life and impaired functioning (12–16). Cannabis users may experience financial problems; notably, a dose–response relationship has been observed between the severity of CUD and workplace absenteeism (17). Cannabis use can also provoke transient psychotic symptoms and an increased risk of developing mental disorders (e.g., psychotic disorder, bipolar disorder, depression), resulting in increased psychiatric service use and hospitalizations. An association was also observed with violent behaviors, both self-directed and toward others (e.g., physical aggression, suicide attempts, completed suicide) (18–22). Furthermore, healthcare costs associated with cannabis use are considerable, and the impacts on hospitals and health and social services have increased over the years, notably due to longer and more frequent hospital stays (23, 24). Given the wide range of negative impacts associated with cannabis use on the individual, their family and peers, as well as on society, effective treatments for problematic cannabis use are critical.

Currently, no pharmacological treatment for cannabis use has received approval, largely due to the limited evidence supporting the efficacy and safety of a few potential treatments (25–27). This lack of pharmacological options underscores the importance of psychosocial interventions, which remain the first line of treatment for cannabis use (28). The psychological interventions recommended for cannabis use are brief interventions, peer support programs, cognitive-behavioral therapy (CBT), motivational approaches (M) such as motivational enhancement therapy and motivational interviewing, contingency management (CM), including voucher-based incentives and contingency management, and a combination of these modalities (29–31). Given the range of available intervention methods, meta-analyses remain among the most valuable research tools, providing a quantitative estimate of effect magnitude based on multiple individual studies (32, 33). However, unlike substances such as alcohol and nicotine, few meta-analytic evidence on the effectiveness of psychotherapy for cannabis use in adults have been undertaken. Indeed, meta-analytic evidence for psychotherapy targeting cannabis use in adults remains limited, heterogeneous across measures and follow-up periods, length of interventions (i.e., a few minutes, several sessions), and in need of updating (34–41). In addition, some meta-analyses have included both studies of people with and without mental disorders (36, 37, 42). Considering that individuals with mental disorders seem not to respond to treatment in the same way as individuals without mental disorders, it is relevant to evaluate efficacy separately (43).

Moreover, heterogeneity is also observed in the type of outcomes. Indeed, clinically and socially relevant outcomes such as cannabis-related problems and cannabis dependence are often overlooked (see **Supplementary Table 1** for a brief summary of measures reported by meta-analyses on the effectiveness of psychological interventions for cannabis use) (35–37, 42). Indeed, cannabis-related problems frequently constitute the main reason for seeking consultation and initiating treatment, notwithstanding the frequency or quantity consumed (44–46). The interindividual variability of cannabis-related negative consequences highlights that measures beyond consumption can better reflect the real-life impact of use on daily functioning (47). Consequently, minimizing the health and social consequences of substance use remains an integral component of many national drug strategies (47–50). By integrating results using both direct comparisons and analyses isolating the effect of one intervention delivered alongside another, it was possible to include a greater number of outcomes in the present meta−analysis. Accordingly, a meta-analysis was conducted to summarize the current state of knowledge on the efficacy of different psychological therapies for cannabis use in adults without severe mental disorders. Since cannabis-related problems often constitute the primary reason for initiating treatment, and there is significant interindividual variability in the impact of cannabis use, the primary outcome was cannabis-related problems. Secondary outcomes comprised the severity of CUD, cannabis consumption patterns (frequency, quantity, and abstinence), and motivation to change cannabis use. The aim is to help guide clinicians in their practice to deliver the highest standards of care for affected individuals.

## 2 Methods

### 2.1 Search strategies

A systematic search was independently conducted by two students (S.G and E.A.) in the electronic databases of PubMed (k= 3,091), PsycINFO (k=2,758), and Web of Science (k=4,977) with keywords that were inclusive for cannabis use (e.g., marijuana, THC) and psychological interventions (e.g., psychotherapy, psychological treatment, CBT, relapse prevention). A secondary search was then conducted in Google Scholar to retrieve grey literature (C = 63), and reference lists of included manuscripts were screened to ensure that no pertinent studies were missed. No setting, date, or geographic restrictions were applied. Searches were limited to English- or French-language sources and were completed on 7 May 2025. A complete electronic search strategy is available in **Supplementary Table 2** in the **Supplementary Material**. This study was not registered in advance, and no prior protocol was published.

### 2.2 Study eligibility

Studies were included for analyses if they met the following criteria: (1) psychosocial intervention that had the goal of targeting specifically cannabis use, (2) quantitatively examined the effects of a psychological therapy on cannabis-related problems, severity of dependence, consumption of cannabis (e.g., abstinence, quantity, frequency), motivation to change cannabis use, quality of life, or functioning, (3) compared psychological intervention to a control group (e.g., other psychological intervention, waiting list, TAU), and (4) the sample included individuals aged 18 years or older. To maximize inclusion, no restrictions about a met diagnostic of CUD or minimal cannabis use, seeking treatment for their cannabis use, or using other drugs. Studies were excluded if they (1) did not include the presence of a healthcare professional (i.e., computer-based intervention, self-help), (2) had as an inclusion criterion the presence of a mental disorder other than a substance use disorder, (3) comprised two sessions or less, which was considered as a brief intervention (39, 41, 51), as several recent meta-analysis have already addressed this type of intervention (38, 39, 52), (4) comprised an individual in control environments (ex., correctional setting), and (5) included trial of pharmacotherapy for the treatment of cannabis use. To maximize the number of studies and obtain an overall view of the subject, quasi-experimental studies were included alongside clinical trials (e.g., randomized controlled trials (RCTs)). Study eligibility was assessed independently by S.G. and E.A., and discussions regarding inclusion of meta-analyses were held with senior researchers (A.D. and S.P.) to ensure consensus. The exclusion criteria were defined to allow the comparison of intervention effectiveness according to the setting, the intervention modalities, and the populations, which, based on the literature, may differ substantially. For example, controlled settings (e.g., detention facilities, psychiatric hospitals, and detoxification centers) were not included and compared to interventions in the community because access to substances is restricted in such environments. Moreover, self-reported substance use could be biased in closed settings due to prohibitions and potential consequences associated with substance use.

### 2.3 Data extraction

Data were extracted with a standardized form by S.G. and counter-validated by D.D., L.K.F., and S.M., including the design of studies, types of psychological intervention, country of study, participants’ characteristics, control group, time points, outcomes measured, and study results (see **Supplementary Table 3** in the **Supplementary Materials**). The authors were contacted when data were missing to perform our analyses or when the condition of a group was unclear. In the absence of a response from the authors, their results were excluded from the analysis. S.G. assessed the risk of bias in five domains (i.e., randomization process, deviations from intended interventions, missing outcome data, measurement of the outcome and selection of the reported result) with the RoB 2 tool for randomized trial Studies were assigned to the following overall risk of bias categories: low risk of bias, some concerns, and high risk of bias. To achieve a high standard of reporting, we followed the Preferred Reporting Items for Systematic Reviews and Meta-Analyses (PRISMA) guidelines (see **Supplementary Table 4**) (53).

### 2.4 Statistical analysis

The analyses were performed using the statistical software RStudio (version 2024.12.1) with the metafor package (54). To assess the specific effects of interventions and maximize the number of available outcomes, results were combined using two approaches. First, outcomes were included when the intervention of interest was directly compared with other interventions. Second, comparisons of a single intervention and its combination with another were also included. Due to the limited number of studies, all analyses were conducted by categorizing control groups as either active or inactive. An active control group was defined as an intervention with a therapeutic objective related to cannabis use. In contrast, an inactive control group was defined as aiming to control for non-specific factors (e.g., treatment-as-usual, delayed intervention, or case management without skills relevant to managing substance use). The time points were grouped as follows: post intervention],1 to 3-month]],3 to 6-month]],6 to 9-month]],9 to 12-month], and [12-month or more after the end of the intervention. This grouping maximized the use of available outcome data across the included studies. Effect sizes were estimated using standardized mean differences (SMDs) for continuous outcomes and odds ratios (ORs) for dichotomous outcomes. The effect sizes provided by SMD were categorized as: small (SMD = 0.20-0.49), medium (SMD = 0.5-0.79), and large (SMD≥ 0.8), and the association provided by OR as: weak (OR = 1.68–3.46), moderate (OR = 3.47–6.70), and strong (OR≥6.71) (55, 56). For all studies, confidence intervals (CIs) were calculated from post-treatment score differences between the experimental and control groups. A confidence interval that crosses zero for SMDs indicates that the effect is not statistically significant. For ORs, a confidence interval that crosses 1 likewise reflects a non−significant effect. Random-effects models were employed, which are more conservative than fixed-effects models and appear to account for heterogeneity across studies and study samples (57). Heterogeneity among study point estimates was quantified using the I^2^ index, with a value of 0% indicating no effect heterogeneity. Values of 25%, 50%, and 75% correspond to low, moderate, and high heterogeneities, respectively (58). The risk of publication bias was assessed using Egger’s test and examined with funnel plots. A significant p-value suggests the presence of publication bias, indicating that studies with non-significant results are more likely to remain unpublished (59). Due to the limited number of studies in each analysis, publication bias was assessed using the pooled dataset across all outcomes. Analyses were performed by S.G. and validated by a statistician (C.-É. G.).

## 3 Results

### 3.1 Description of studies

**Figure 1:**
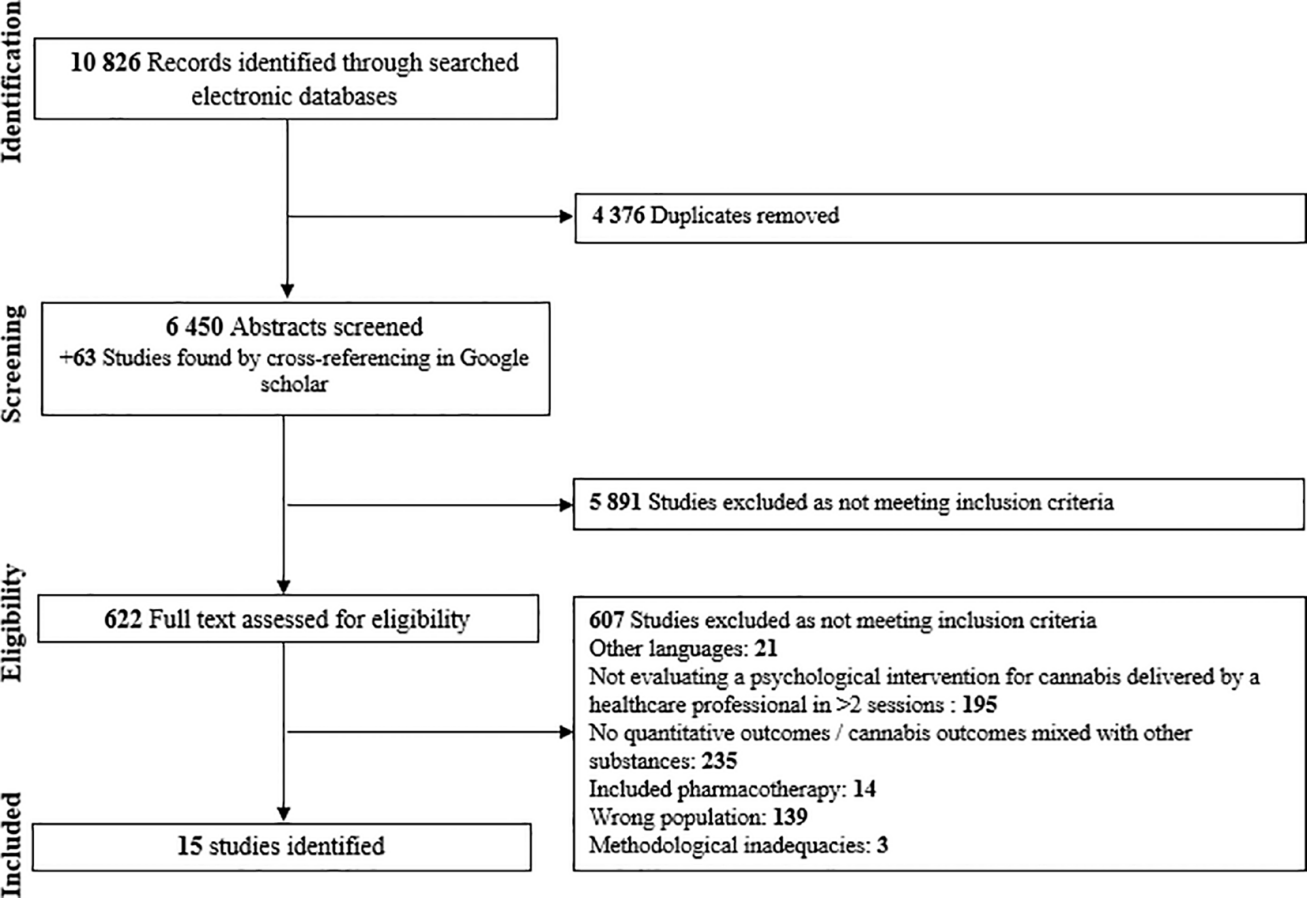
Flow-chart depicting the search strategy employed to find the studies to include in the meta-analysis.

The systematic search identified 6,450 potential articles, which were screened for eligibility after duplicates were removed. Among these, 15 studies were included. The interventions examined comprised CBT, M, CM, drug counseling (DC), and their combination, as well as relapse prevention, social support group, mindfulness, and community reinforcement. The PRISMA flowchart for study inclusion in the meta-analyses is shown in **Figure 1**. Regarding study designs, two randomized trials and thirteen randomized controlled trials were retrieved; eight were rated as having some concerns and seven as having a high risk of bias according to RoB 2. Refer to **Supplementary Table 3** for a summary of the risk of bias provided. Most studies were conducted in the United States of America (C = 10), with additional studies from Germany (C = 2), Australia (C = 1), Brazil (C = 1), and France (C = 1). Below, we examine the effects of the therapeutic approaches on the consequences of cannabis use (e.g., severity of cannabis use disorder, cannabis-related problems) as well as on consumption of cannabis (i.e., abstinence, frequency, quantity).

### 3.2 Effects of psychological interventions

#### 3.2.1 Cognitive behavioral therapy

CBT has been evaluated in four studies, totaling six types of comparisons between two groups [number of different comparison groups (C)] and including 677 participants (60–63). The comparison groups comprised: 1) CBT compared to CM, 2) CBT+CM versus CM alone, 3) CBT+M versus a brief intervention, 4) CBT+M versus a M alone, 5) CBT+social support group versus a brief intervention, and 6) CBT versus a delayed treatment control. Of the studies included, three were rated as having a high risk of bias, and one was rated as having some concerns.

**Figure 2:**
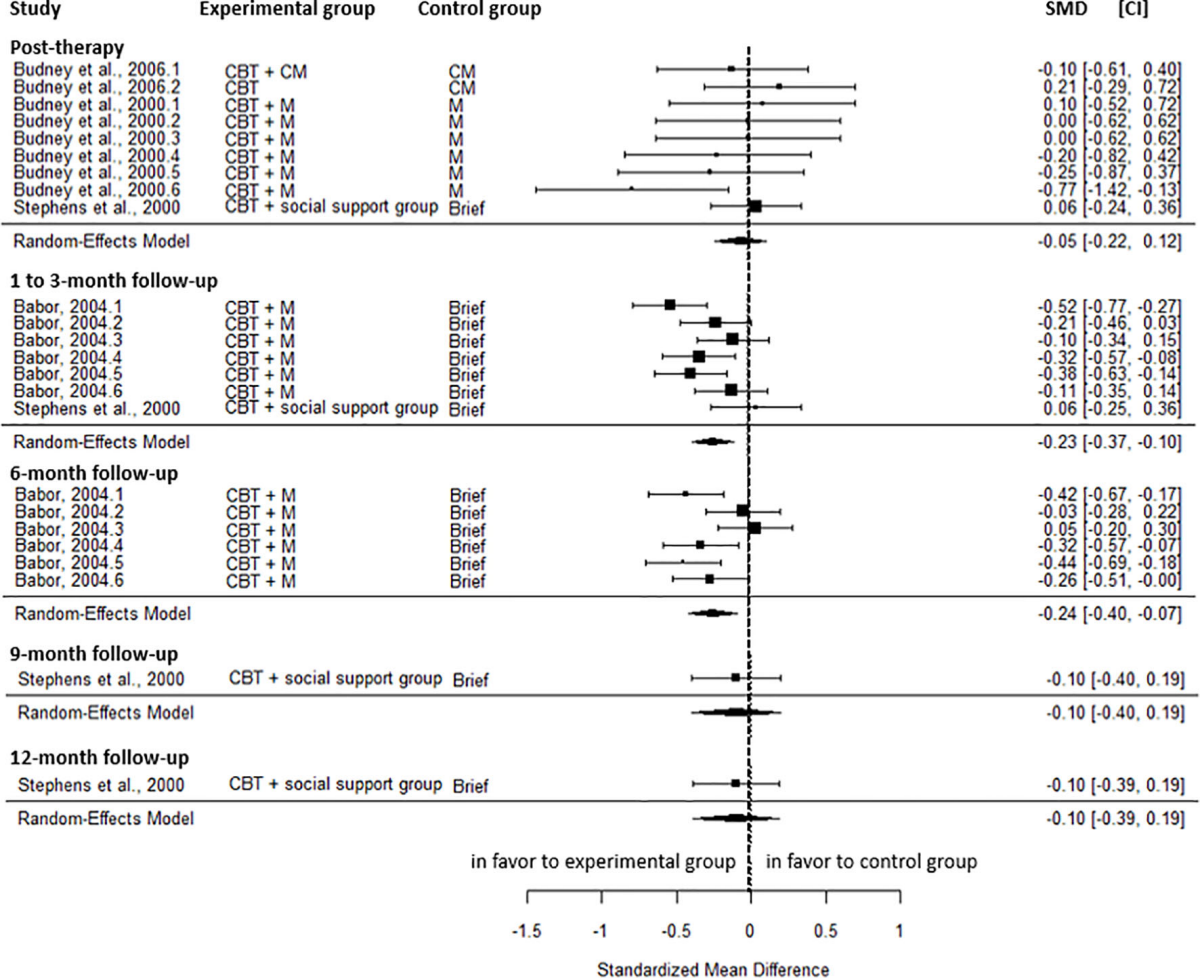
Effect sizes of CBT on cannabis-related problems compared to active control at post-intervention, 1 to 3-, 6-, 9-, and 12-month follow-up. CM, contingency management; CBT, cognitive behavioral therapy; M, motivational approach; Brief, brief intervention; SMD, standardized mean differences; CI, confidence interval.

Results showed no significant post-intervention differences in cannabis-related problems between CBT and the active control group (SMD=-0.05, CI=-0.22; 0.12, C = 4), with no heterogeneity (I^2^ = 0.00%). However, a small reduction was observed in favor of CBT at the 1 to 3-month follow-up (SMD=-0.23, CI=-0.37; -0.10, C = 2), and the 6-month follow-up (SMD=-0.24, CI=-0.40; -0.07, C = 1). Moderate heterogeneity was observed for both results (I² = 52.88% and 59.25%). No significant differences were found at the 9-month (SMD=-0.10, CI=-0.40; 0.19, C = 1) and the 12-month follow-up (SMD=-0.10, CI=-0.39; 0.19, C = 1) (**Figure 2**).

The severity of cannabis dependence was not significantly different between the two groups at post-intervention (SMD = 0.01, CI=-0.30; 0.31, C = 1), at the 1 to 3-month (SMD=-0.19, CI=-0.87; 0.49, C = 2, I^2^ = 91.71%), the 9-month (SMD = 0.00, CI=-0.29; 0.30, C = 1), and the 12-month follow-up (SMD = 0.02, CI=-0.27; 0.32, C = 1). However, a small, significant reduction in the severity of cannabis dependence was observed at the 6-month follow-up (SMD=-0.36, CI=-0.62, -0.11, C = 1). At post-intervention, no significant difference was found in the frequency of cannabis use (SMD=-0.16, CI=-0.39; 0.07, C = 3, I^2^ = 0.00%). A small reduction in the frequency of use was observed at the 1 to 3-month (SMD=-0.29, CI=-0.58; 0.00, C = 4, I^2^ = 59.62%) and the 6-month (SMD=-0.42, CI=-0.63; -0.21, C = 3, I^2^ = 0.00%) in favor of CBT (intervention group) over the active control group. The results were not maintained at the 9-month (SMD=-0.19, CI=-0.53; 0.15, C = 3, I^2^ = 47.19%) and at 12-month follow-up (SMD=-0.16, CI=-0.33; 0.01, C = 4, I^2^ = 0.00%). Regarding the quantity of cannabis use, no significant differences were found at post-intervention (SMD=-0.22, CI=-0.71; 0.28, C = 2, I^2^ = 47.20%) and the 6-month follow-up (SMD=-0.05, CI=-0.30, 0.20, C = 1). At 1-month, a small significant reduction in the quantity of consumption (SMD=-0.30, CI=-0.54; -0.06; C = 1) was found in favor of CBT compared to the active control group. At post-intervention, a moderate effect was observed on readiness to change cannabis use (SMD = 0.79, CI = 0.15; 1.44, C = 1) in favor of CBT compared to the active group. The odds of abstinence did not differ significantly between the CBT and the active control groups at post-intervention (OR = 0.87, CI = 0.41; 1.82, C = 2, I^2^ = 0.00%), the 6-month (OR = 1.35, CI = 0.76; 2.42, C = 3, I^2^ = 2.73%), and the 9-month follow-up (OR = 1.47, CI = 0.47; 4.64, C = 2, I^2^ = 33.89%). CBT was associated with a weak increase in the odds of abstinence compared to the active control group at the 1 to 3-month (OR = 2.71, CI = 1.54; 4.77, C = 3, I^2^ = 0.00%) and the 12-month follow-up (OR = 2.04, CI = 1.20; 3.46, C = 2, I^2^ = 0.00%).

At post-intervention, compared to inactive control, CBT showed a large reduction in frequency of cannabis use (SMD=-1.01; CI=-1.33; -0.69, C = 1), cannabis related-problem (SMD=-1.03; CI=-1.35; -0.72, C = 1), and severity of cannabis dependence (SMD=-1.00, CI=-1.31; -0.68, C = 1).

In summary, CBT compared to active control showed small benefits on the cannabis-related problems, severity of cannabis dependence, frequency and quantity of cannabis use, and abstinence, and moderate benefits on motivation to change at specific time points. However, the majority of comparisons were non-significant and most of the statistically significant findings were based on a single study. At post-intervention, when compared with inactive control groups, CBT showed large reductions in cannabis-related problems, severity of dependence, and frequency of cannabis use. These results were from a single study.

#### 3.2.2 Contingency management

CM has been evaluated in six studies, totaling seven types of comparisons and including 756 participants (61, 62, 64–67). The comparison groups comprised: 1) CM compared to CBT, 2) CBT+CM compared to CBT alone, 3) CM compared to CBT+M, 4) CBT+M+CM compared to CBT+M, 5) CM+DC versus DC, 6) CM+M compared to M alone, and 7) CM compared to case management without components for substance use. Of the included studies, three were rated as having a high risk of bias, and three were rated as having some concerns.

**Figure 3:**
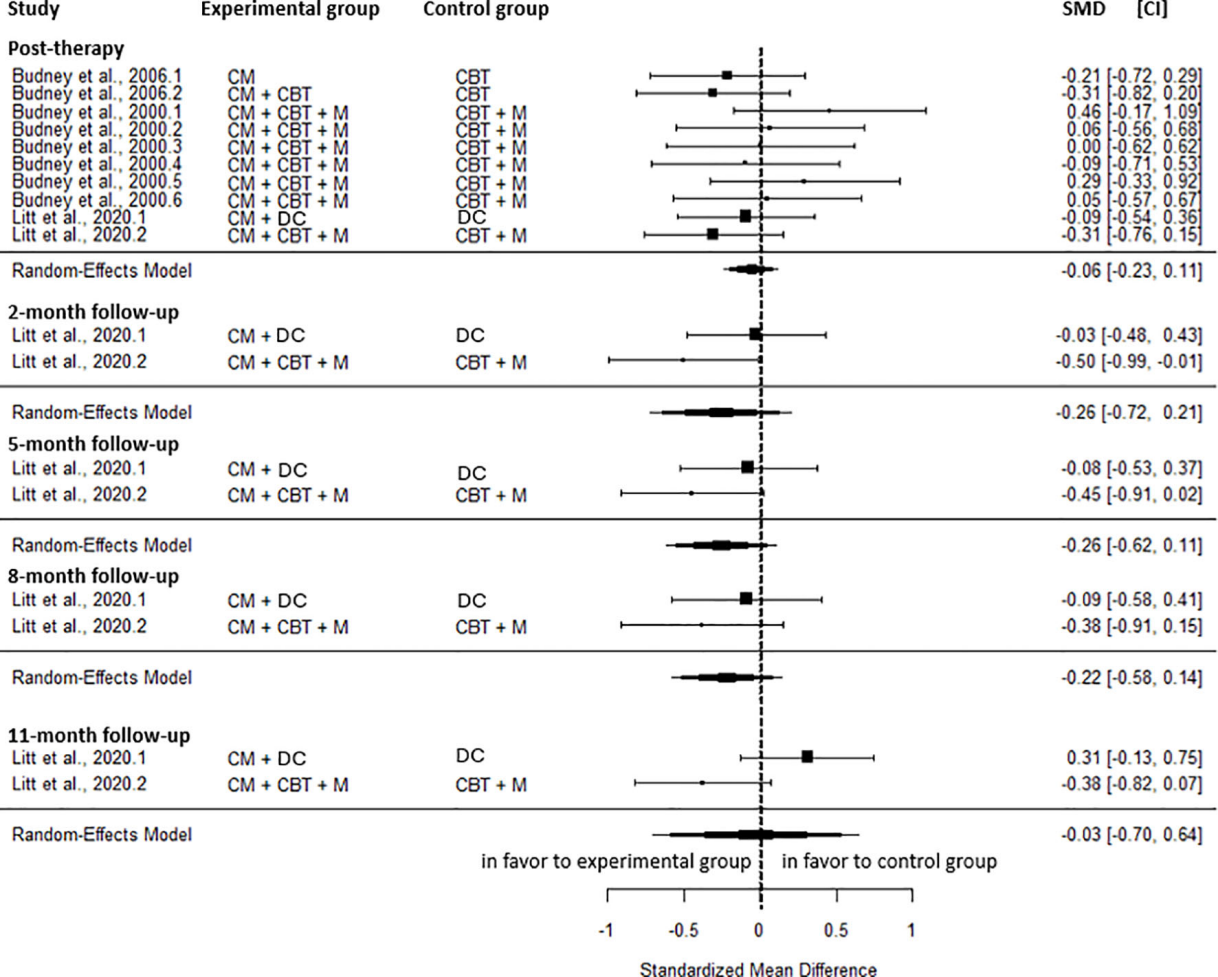
Effect sizes of CM on cannabis-related problems compared to active control at post-intervention, 2-, 5-, 8, and 11-month follow-up. CM, contingency management; CBT, cognitive behavioral therapy; M, motivational approach; DC, drug counseling; SMD, standard mean deviation; CI, confidence interval.

No significant differences were observed between CM and the active control for cannabis-related problems at any time point, including the post-intervention (SMD=-0.06, CI=-0.23; 0.11, C = 10, I^2^ = 0.00%), the 2-month (SMD=-0.26, CI=-0.72; 0.21, C = 2, I^2^ = 48.31%), the 5-month (SMD=-0.26, CI=-0.62; 0.11, C = 2, I^2^ = 20.02%), the 8-month (SMD=-0.22, CI=-0.58; 0.14, C = 2, I^2^ = 0.00%), and the 11-month follow-up (SMD=-0.03, CI=-0.70; 0.64, C = 2, I^2^ = 78.37%) (**Figure 3**).

The severity of cannabis dependence did not differ significantly between CM and active control groups at either the post-intervention (SMD = 0.18, CI=-0.32; 0.67, C = 1) or the 1-month follow-up (SMD = 0.11, CI=-0.38; 0.60, C = 1). At post-intervention, no significant differences were observed between CM and the active control in measures of readiness to change cannabis use (SMD = 0.08, CI=-0.14; 0.30, C = 4), self-efficacy (SMD=-0.18, CI=-0.47; 0.11, C = 2), or coping skills (SMD = 0.04, CI=-0.23; 0.30, C = 2). No heterogeneity was found for readiness to change cannabis use or coping skills, whereas low heterogeneity was observed for self-efficacy (I²=14.81%). At post-intervention, a small reduction in the frequency of cannabis use was found (SMD = 0.30, CI = 0.01; 0.59, C = 2, I^2^ = 0.00%) in favor of active control compared to CM. This effect was not sustained at subsequent follow-up assessments, including 1 to 3-month (SMD = 0.15, -0.14; 0.44, C = 3, I^2^ = 0.00%), 6-month (SMD=-0.08, CI=-0.39; 0.54, C = 2, I^2^ = 40.65%), 9-month (SMD=-0.09, CI=-0.62; 0.44, C = 2), and 12-month post-intervention (SMD=-0.20, CI=-0.56; 0.17, C = 2, I^2^ = 3.09%). At post-intervention, the active control group showed a small, significant decrease in the quantity of cannabis use (SMD = 0.46, CI = 0.10; 0.82, C = 2) over the CM. There was no heterogeneity. At post-intervention, CM was associated with a weak increase in the odds of abstinence (OR = 1.70, CI = 1.01; 2,88, C = 4) compared to the active control group. There was no heterogeneity. However, no statistically significant differences on abstinence were observed at subsequent follow-up; 3-month (OR = 1.72, CI = 0.59; 7.38, C = 5, I^2^ = 0.00%), 5 to 6-month (OR = 1,06, CI = 0.64; 1.76, C = 6, I^2^ = 21.60%), 8-to-9 month (OR = 1.29, CI = 0.69; 2.42, C = 4, I^2^ = 16.83%), 11-months follow-up (OR = 1.26, CI = 0.72; 2.29, C = 4, I^2^ = 0.00%) and 14-month (OR = 0.95, CI = 0.37; 2.46, C = 2, I^2^ = 43.06%). In another study, no significant results were observed for the proportion of days participants reported being abstinent at post-intervention (SMD = 0.20, CI=-0.29; 0.70, C = 2) neither at the 2-month follow-up (SMD = 0.25, CI=-0.34; 0.83, C = 2), the 5-months follow-up (SMD = 0.21, CI=-0.34; 0.76, C = 2), at the 8-month follow-up (SMD = 0.03, CI=-0.45; 0.51, C = 2), and the 11-month follow-up (SMD = 0.09, CI=-0.32; 0.50, C = 2). These results showed moderate heterogeneity. At post-intervention, no significant differences were observed between CM and the inactive control group in readiness to change cannabis use (SMD = 0.35, CI=-0.04; 0.74, C = 1), self-efficacy (SMD = 0.21, CI=-0.18; 0.59, C = 1), or coping skills (SMD = 0.02, CI=-0.37; 0.40, C = 1). In addition, compared to the inactive control condition, CM did not differ significantly in the odds of abstinence at post-intervention. (OR = 2.26, CI = 0.77; 6.65, C = 1), the 5-month (OR = 1.53, CI = 0.53; 4.36, C = 1), the 8-month (OR = 0.78, CI = 0.25; 2.45, C = 1), the 11-month (OR = 0.79, CI = 0.25; 2.46, C = 1), and the 14-month follow-up (OR = 0.60, CI = 0.20; 1.80, C = 1). In summary, no significant differences were observed between CM and active control groups at any time point for cannabis-related problems, severity of cannabis dependence, coping skills, or readiness to change cannabis use. A small reduction in the quantity of cannabis use was observed at post-intervention in favor of the active control group. However, this outcome was not assessed at subsequent follow-up points. At post-intervention, a small decrease in frequency of use and a weak increase in odds of abstinence were observed in favor of the active control group. Nevertheless, these effects were not maintained at subsequent follow-up. No significant differences were observed for readiness to change cannabis use, coping skills, or abstinence when CM was compared with inactive control groups.

#### 3.2.3 Relapse prevention and social support group

The comparison between relapse prevention and the social support group was evaluated in a study involving 167 individuals (68). This study was rated as having a high risk of bias.

The severity of cannabis dependence did not differ significantly between groups at any time point: 3-month (SMD=-0.12, CI=-0.43; 0.20, C = 1), 6-month (SMD = 0.02, CI=-0.30; 0.33, C = 1), and 12-month follow-up (SMD = 0.08, CI=-0.23; 0.40). No significant effects were found for cannabis use frequency across follow-up between the two groups: 1-month (SMD=-0.30, CI=-0.60; 0.01, C = 1), 3-month (SMD=-0.06, CI=-0.36; 0.24, C = 1), 6-month (SMD=-0.01, CI=-0.32; 0.29, C = 1), 9-month (SMD = 0.12, CI=-0.18; 0.43, C = 1), and 12-month (SMD = 0.04, CI=-0.26; 0.34, C = 1). Results showed no significant difference in the odds of abstinence for relapse prevention compared with the social support control group at the 3-month (OR = 0.72, CI = 0.38; 1.35, C = 1), the 6-month (OR = 0.81, CI = 0.40; 1.64, C = 1), and the 12-month follow-up (OR = 0.78, CI = 0.35; 1.78, C = 1).

In summary, relapse prevention and the social support group did not differ significantly from one another for severity of cannabis dependence, frequency of cannabis use, and abstinence.

#### 3.2.4 Mindfulness

Mindfulness was compared with treatment as usual (TAU) in one study, including twenty-eight individuals (69). This study was rated as having a high risk of bias.

Regarding withdrawal symptoms, a large reduction was found at post-intervention (SMD=-1.84, CI=-2.73; -0.95, C = 1); however, this effect was not maintained at the 1-month follow-up (SMD=-1.16, CI=-2.36; 0.04, C = 1). A moderate reduction in the frequency of cannabis use was observed at post-intervention (SMD = 0.79, CI=-1.75, -0.02, C = 1), and a large decrease at the 1-month follow-up (SMD=-1.37, CI=-2.60; -0.13, C = 1). These results should be interpreted with caution, as the reported interquartile data were converted into standard deviations for analysis. The authors indicated that the results were not statistically significant.

#### 3.2.5 Drug counseling

Across three studies, DC was evaluated in six different types of comparisons, involving 386 participants (65, 66, 70). The comparison groups comprised: 1) DC compared to CBT+M, 2) DC compared to CBT+M+CM, 3) DC+CM compared to CBT+M+CM. Of the included studies, one was rated as having a high risk of bias, and two were rated as having some concerns.

**Figure 4:**
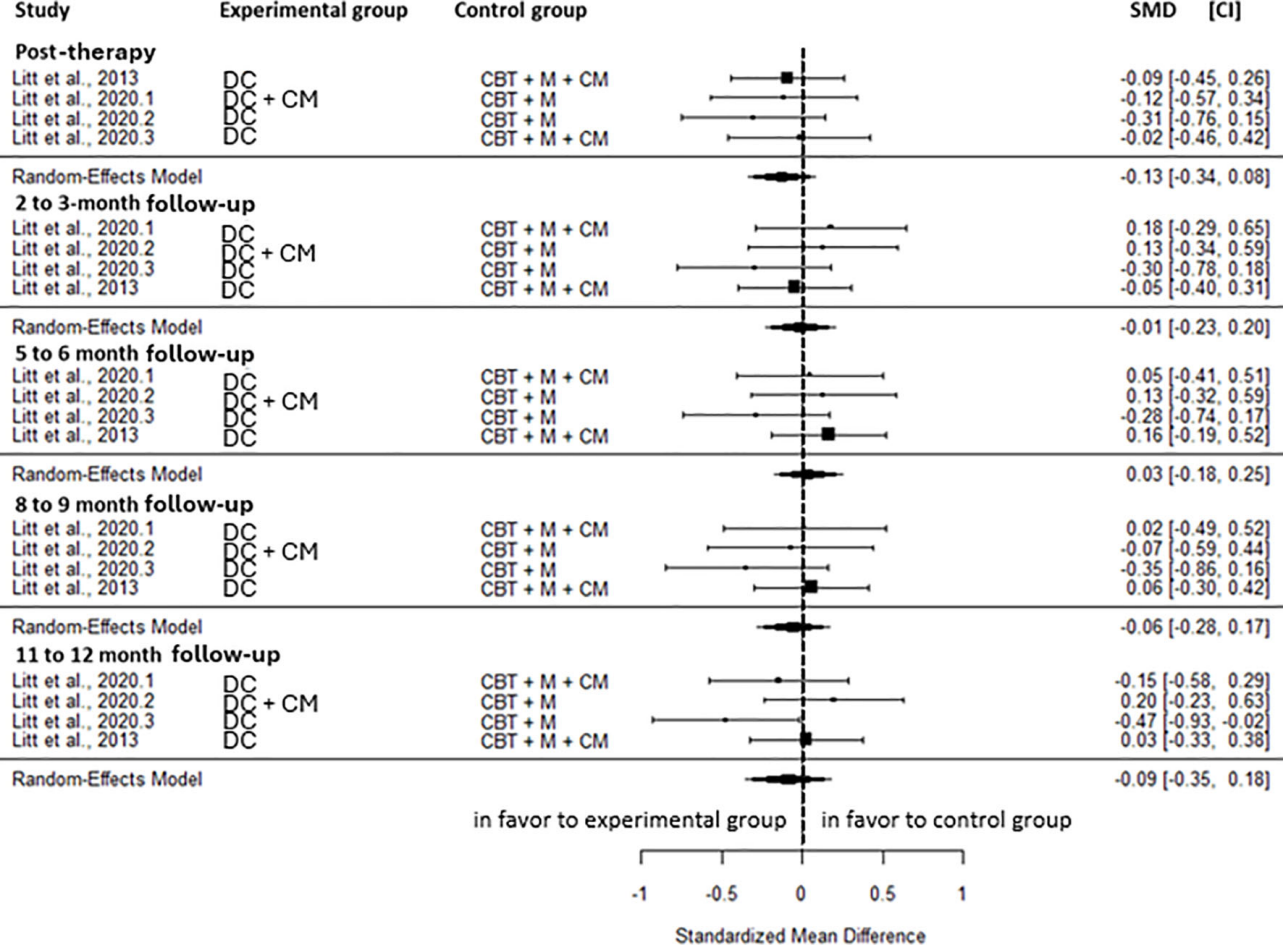
Effect size of drug counseling on cannabis-related problems compared to active control at post-intervention, 2 to 3-, 5 to 6-, 8 to 9-, and 11 to 12-month follow-up. CM, contingency management; CBT, cognitive behavioral therapy; M, motivational approach; DC, drug counseling; SMD, standard mean deviation; CI, confidence interval.

No significant differences were observed between DC and the active control group for cannabis-related problems at any time point; the post-intervention (SMD=-0.13, CI=-0.34; 0.08, C = 4), 2 to 3-month (SMD=-0.01, CI=-0.23; 0.20, C = 4), 5 to 6-month (SMD = 0.03, CI=-0.18; 0.25, C = 4), 8 to 9-month (SMD=-0.06, CI=-0.28; 0.17, C = 4) and 11 to 12-month follow-up (SMD=-0.09; -0.35; 0.18, C = 4) (**Figure 4**). No heterogeneity was observed across all time points, except a low heterogeneity at the 11–12-month follow-up (I^2^ = 37.74%).

Regarding abstinence, no significant difference between DC and active control group was observed at post-intervention (OR = 0.65, CI = 0.23; 1.83, C = 1), the 3-month (OR = 0.44, CI = 0.25; 0.79, C = 4, I^2^ = 0.00%), the 6 month (OR = 0.62, CI = 0.37; 1.01, C = 4, I^2^ = 0.00%), the 9-month (OR = 0.81, CI = 0.35; 1.89, C = 1), and the 12-month follow-up (OR = 0.57, CI = 0.23; 1.44, C = 1). In addition, no significant difference between group was found for the proportion of days being abstinent at any time points; post-intervention (SMD=-0.05, CI=-0.42; 0.32, C = 4, I^2^ = 71.16%), 2 to 3-month (SMD = 0.07, CI=-0.34; 0.48, C = 4, I^2^ = 75.87%), 5 to 6-month (SMD=-0.09, CI=-0.43; 0.35, C = 4, I^2^ = 63.99%), 8 to 9-month (SMD = 0.21, CI=-0.04; 0.46, C = 4, I^2^ = 31.45%), and 11 to 12-month follow-up (SMD = 0.09, CI=-0.16; 0.33, C = 4, I^2^ = 30.19%).

In summary, no significant differences were found between the DC and active control groups for cannabis-related problems, the odds of abstinence, and the proportion of abstinent at any time points.

#### 3.2.6 Cognitive behavioral therapy plus motivational approach

CBT+M has been evaluated in seven studies, totaling 13 types of comparisons and including 1,281 participants (60, 64–66, 71–73). The comparison groups comprised: 1) CBT+M compared to CM, 2) CBT+M compared to DC, 3) CBT+M compared to CM+DC, 4) CBT+M+CM compared to CM alone, 5) CBT+M+CM compared to CM+DC, 6) CBT+M compared to delayed treatment, and 7) CBT+M compared to case management without components of substance use. Of the included studies, two were rated as having a high risk of bias, and five were rated as having some concerns.

**Figure 5:**
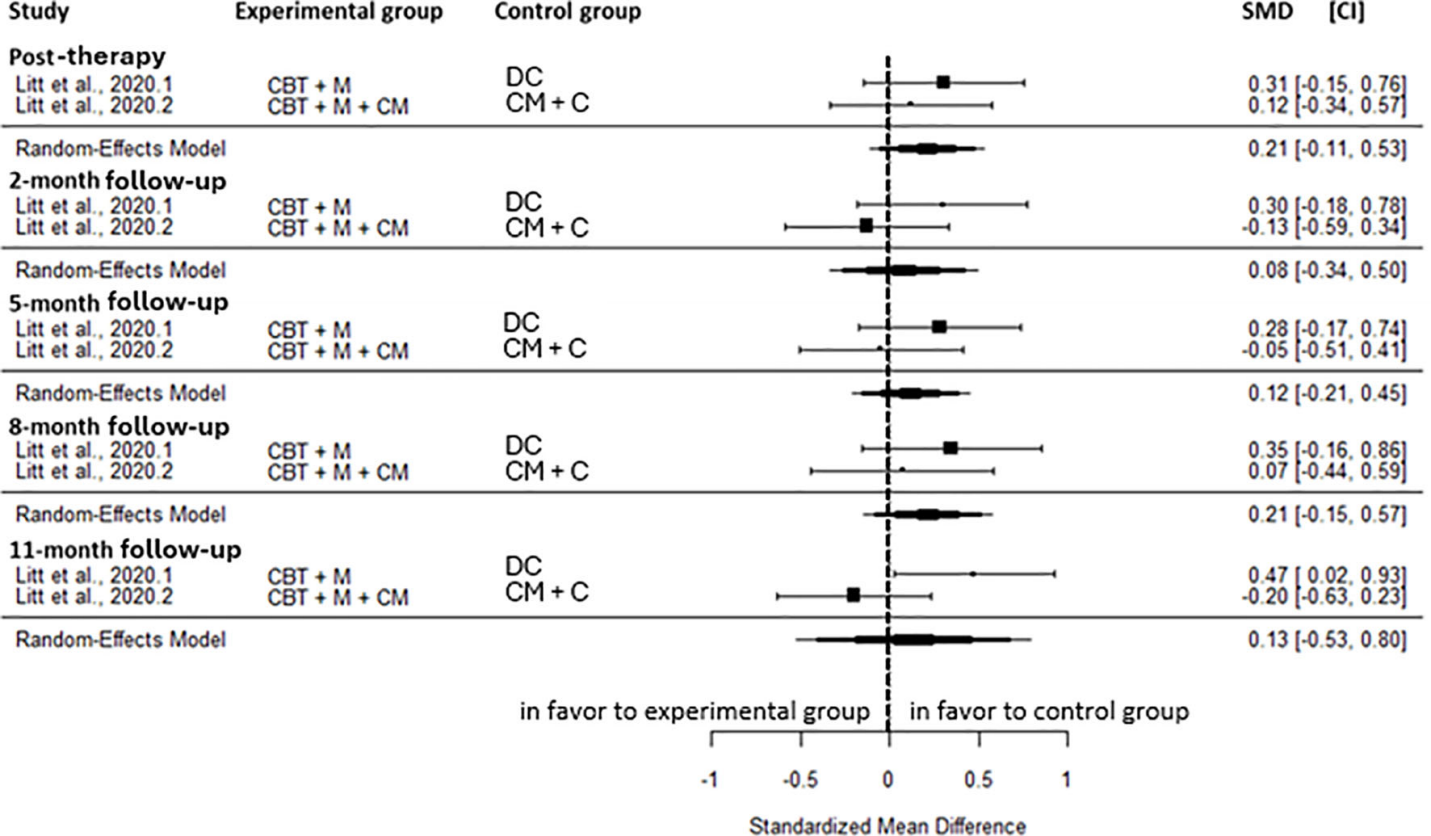
Effect size of cognitive behavioral therapy plus motivational approach on cannabis-related problems compared to active control at post-intervention, 2-, 5-, 8, and 11-month follow-up. CM, contingency management; CBT, cognitive behavioral therapy; M, motivational approach; DC, drug counseling; SMD, standard mean deviation; CI, confidence interval.

Cannabis-related problems did not differ significantly between CBT+M compared to active control groups at any time points; post-intervention (SMD = 0.21, CI=-0.11; 0.53, C = 2, I^2^ = 0.00%), 2-month (SMD = 0.08, CI=-0.34; 0.50, C = 2, I^2^ = 35.89%), 5-month (SMD = 0.12, CI=-0.21; 0.45, C = 2, I^2^ = 1.87%), 8-month (SMD = 0.21, CI=-0.15; 0.57, C = 2, I^2^ = 0.00%) and 11-month follow-up (SMD = 0.13, CI=-0.53; 0.80, C = 2, I^2^ = 77.47%) (**Figure 5**).

At post-intervention, readiness to change cannabis use (SMD=-0.04, CI=-0.31; 0.23, C = 2, I^2^ = 0.00%), self-efficacy (SMD=-0.14, CI=-0.47; 0.19, C = 2, I^2^ = 33.51%) and coping skills (SMD = 0.13, CI-0.14; 0.40, C = 2, I^2^ = 0.00%) were not significant different between the two group. The odds of abstinence did not differ between the CBT+M group and the active control group at post-intervention (OR = 0.66, CI = 0.33; 1.33, C = 2, I2 = 0.00%), the 3-month (OR = 1.46, CI = 0.67; 3.18, C = 3), the 5-month (OR = 1.31, CI = 0.67; 2.57, C = 2), and the 11-month follow-up (OR = 1.79, CI = 0.83; 3.87, C = 2). The odds of abstinence were weak at the 6 to 8-month (OR = 1.89, CI = 1.15; 3.09, C = 5) and the 14-month (OR = 2.20, CI = 1.30; 5.40, C = 2) in favor of CBT+M. All the time points showed no heterogeneity. Regarding the proportion of days abstinent, no significant differences between the two groups were found at post-intervention (SMD=-0.24, CI=-0.54; 0.07, C = 3) and the 5-month follow-up (SMD=-0.17, CI=-0.51; 0.17, C = 3), with low heterogeneity at both time points (I²=39.01% and 47.96%, respectively). However, the active control group was associated with small reductions in the proportion of days abstinent at the 2-month (SMD=-0.40, CI=-0.77; -0.02, C = 3, I^2^ = 57.69%), the 8-month (SMD=-0.32, CI=-0.61; -0.04, C = 3, I^2^ = 22.54%), and the 11-month (SMD=-0.26, CI=-0.51; 0.00, C = 3, I^2^ = 0.00%) compared with CBT+M.

**Figure 6:**
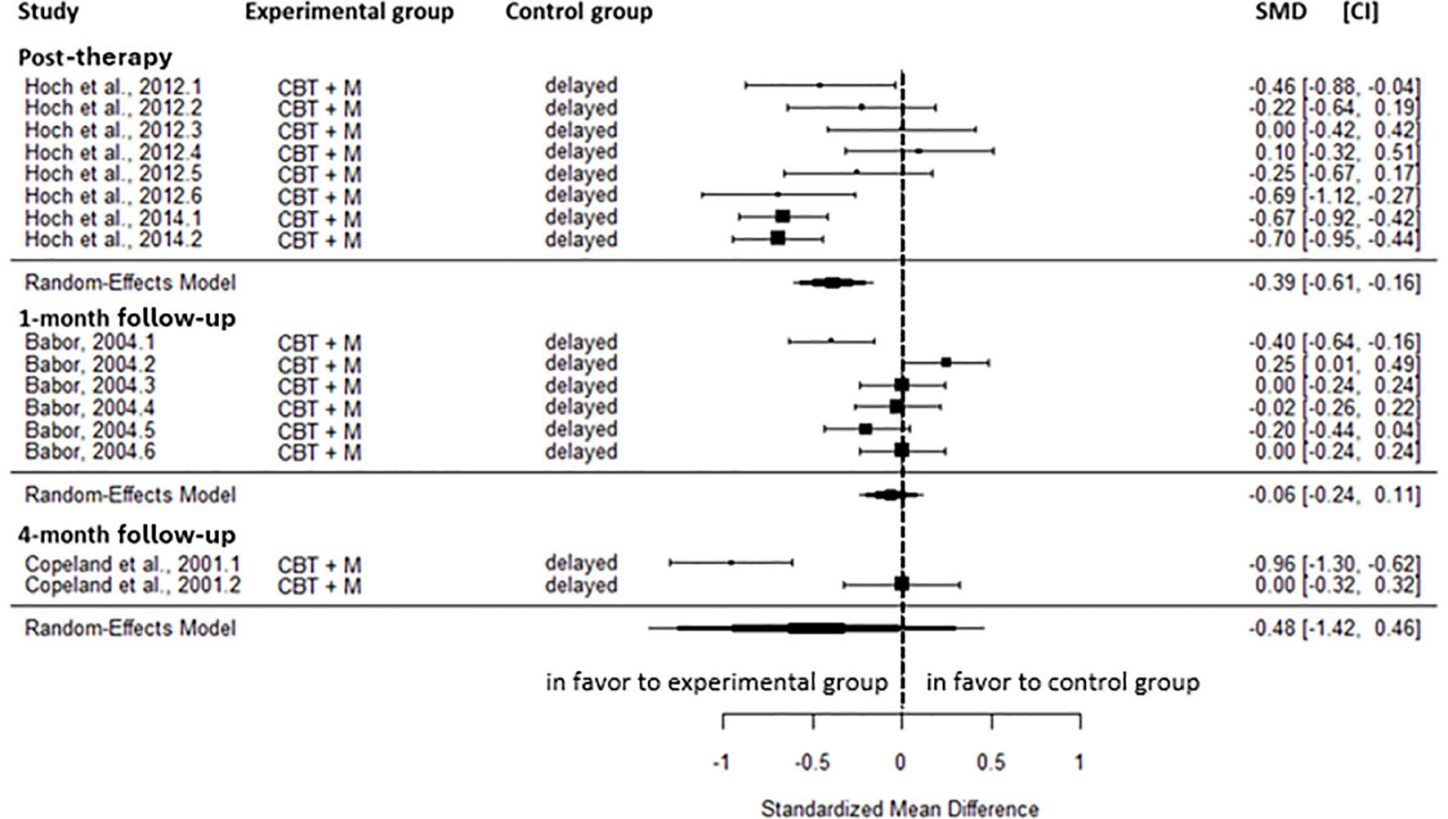
Effect size of cannabis-related problems comparing CBT plus motivational approach to inactive control groups at post-intervention, 1- and 4-month follow-up. CBT, cognitive behavioral therapy; M, motivational approach; SMD, standard mean deviation; CI, confidence interval.

Compared to the inactive control group, a small reduction was found for cannabis-related problems in favor of CBT+M at post-intervention (SMD=-0.39, CI=-0.61; -0.16, C = 2). This result showed moderate heterogeneity (I^2^ = 66.83%). However, no significant difference was found at the 1-month (SMD=-0.06, CI=-0.24;0.11, C = 1) and the 4-month follow-up (SMD=-0.48, CI=-1.42; 0.46, CI = 1) (**Figure 6**).

A significant moderate reduction in the severity of cannabis dependence was found at post-intervention (SMD=-0.69, CI=-0.96; -0.42, C = 1) and a large reduction at the 1-month (SMD=-0.88, CI=-1.13; -0.63, C = 1) and the 4-month follow-up (SMD=-0.88, CI=-1.22; -0.55, C = 1) in favor of CBT+M. At the 1-month follow-up, a large effect (SMD=-1.13, CI=-1.38; -0.87, C = 1) was observed in the frequency of cannabis use in favor of CBT+M. Regarding the quantity of cannabis use, a moderate reduction was found at post-intervention (SMD=-0.72, CI=-0.94; -0.50, C = 2, I^2^ = 0.00%), the 1-month (SMD=-0.56, CI=-0.80; -0.32, C = 1), and the 4-month follow-up (SMD=-0.52, CI=-0.85; -0.20, C = 1) in favor of CBT+M. At post-intervention, readiness to change cannabis use, self-efficacy, and coping skills were not significantly different between CBT+M and the inactive control groups. At post-intervention, participants receiving CBT+M had weakly higher odds of abstinence compared with those in the inactive control group (OR = 3.30, CI = 1.38; 7.90, C = 3, I^2^ = 61.94%) and the 1-month follow-up strongly higher odds (OR = 7.76, CI = 2.91; 20.72, C = 1). Results were not significant at the 4 to 5-month (OR = 1.21, CI = 0.67; 2.17, C = 2, I^2^ = 5.71%), the 8-month (OR = 1.28, CI = 0.46; 3.54, C = 1), the 11-month (OR = 1.00, CI = 0.34; 2.90, C = 1) and the 14-month follow-up (OR = 1.28, CI = 0.46; 3.54, C = 1).

In summary, CBT+M showed no significant differences compared with the active control group for cannabis-related problems at short- and medium-term follow-up. Likewise, no significant post-intervention effects were observed for readiness to change, self-efficacy, or coping skills. A small effect on the proportion of days abstinent, along with weak odds of abstinence, favored CBT+M at specific time points between post-intervention and the 14-month follow-up. However, most comparisons were not statistically significant. Compared with inactive control groups, CBT+M produced a small reduction in cannabis-related problems at post-intervention. This effect was not maintained at medium-term follow-up. A moderate and large effect was observed for the severity of cannabis dependence at two different short-term follow-ups. These results were based on a single study. No significant effects were found for readiness to change cannabis use, self-efficacy, or coping skills at post-intervention. A large significant effect was observed for the frequency of use, driven by a single study. These outcomes were not evaluated at subsequent follow-up assessments. A moderate effect was observed on the quantity of use at post-intervention and at the subsequent short-term follow-up. In addition, increased odds of abstinence were observed during short-term follow-up, but these effects were not sustained at subsequent time points.

#### 3.2.7 Cognitive behavioral therapy plus contingency management

CBT+CM has been evaluated in a study involving 40 participants that compared CBT combined with CM to M alone (61). This study was rated as having some concerns regarding bias.

**Figure 7:**
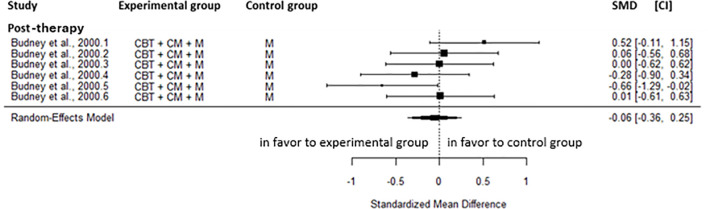
Effect size of cannabis-related problems comparing CBT plus CM to active control groups at post-intervention. CBT, cognitive behavioral therapy; CM, contingency approach; M, motivational approach; SMD, standard mean deviation; CI, confidence interval.

At post-intervention, there was no significant difference in cannabis-related problems (SMD=-0.06; CI=-0.36; 0.25, C = 1) between the two groups (**Figure 7**).

At post-intervention, a moderate and significant difference was observed for readiness to change cannabis use (SMD = 0.69, CI = 0.05; 1.33, C = 1) in favor of CBT+CM compared to the active control.

#### 3.2.8 Motivational approach plus relapse prevention

One study compared M+relapse prevention with delayed treatment, yielding two comparisons and 160 participants (74). This study was rated as having some concerns regarding bias.

**Figure 8:**
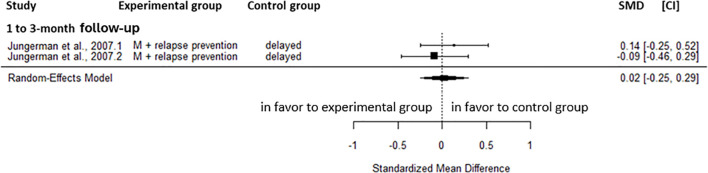
Effect size of cannabis-related problems comparing motivational approach plus relapse prevention to inactive treatment at 1 to 3-month follow-up. M, motivational approach; SMD, standard mean deviation; CI, confidence interval.

At 1 to 3-month follow-up, M+relapse prevention did not significantly impact on cannabis-related problem (SMD = 0.02, CI=-0.25; 0.29, C = 2) compared to delayed treatment (**Figure 8**). These results showed no heterogeneity.

At post-intervention, no significant effect was observed for the severity of cannabis dependence (SMD=-0.24, CI=-0.51; 0.03, C = 2) and the odds of abstinence (OR = 0.99, CI = 0.23; 4.26, C = 2) compared with delayed treatment. A large reduction in the frequency of cannabis (SMD=-0.80, CI=-1.08; -0.52, C = 2) and a moderate decrease in quantity of cannabis use (SMD=-0.60, CI=-0.88; -0.33, C = 2) were found in favor of the M+relapse prevention compared to the inactive control group at the 1 to 3-month follow-up. No heterogeneity was observed for these results.

#### 3.2.9 Contingency management plus drug counselling

CM+DC, has been evaluated in two studies involving 128 participants. These studies include two comparisons of CM+DC versus CBT+M (65, 66). These two studies were rated as having some concerns regarding bias.

**Figure 9:**
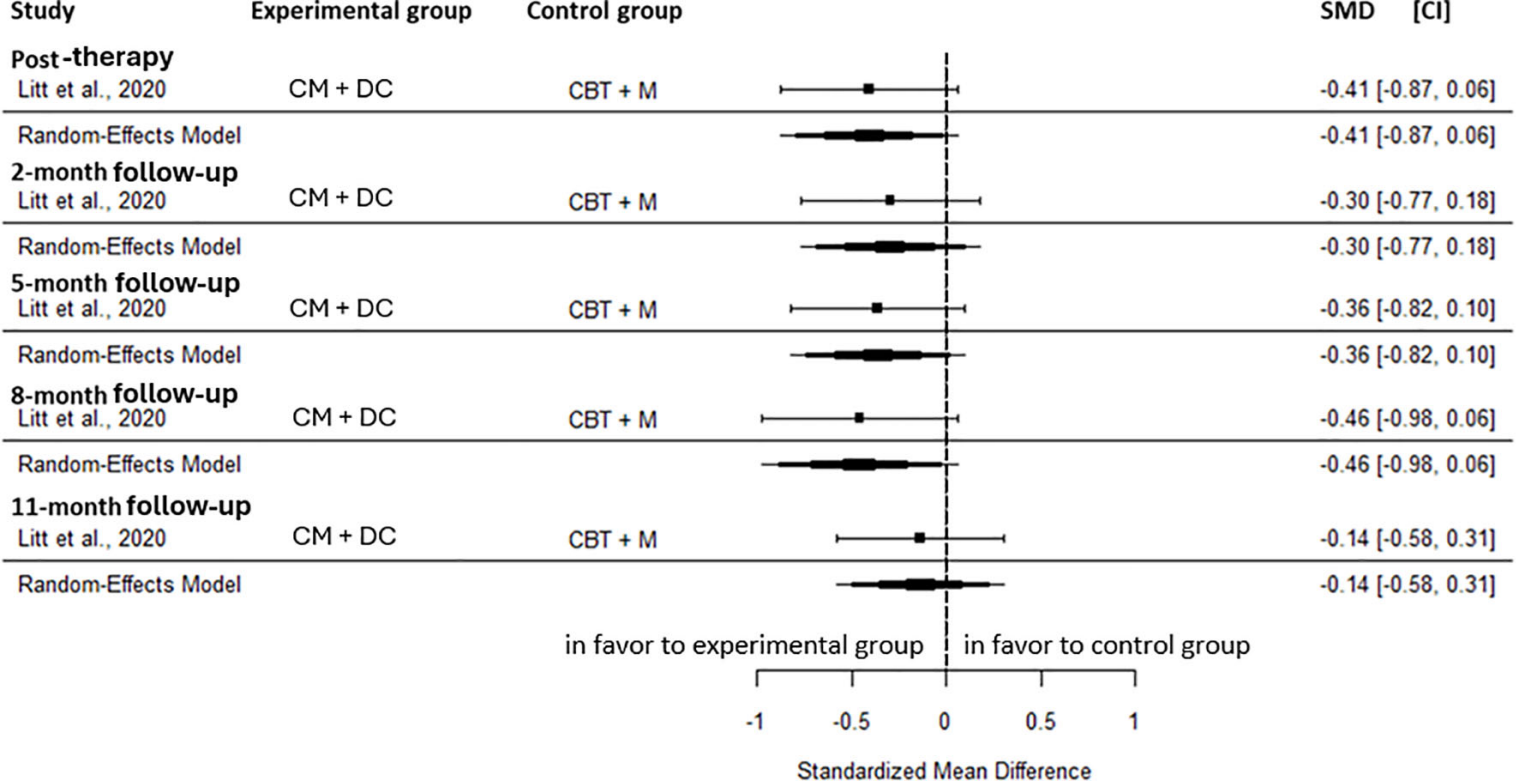
Effect size of cannabis-related problems comparing CM**+**DC to an active control group at post-intervention, 2-, 5-, 8-, and 11-month follow-up. CM: contingency management, CBT, cognitive behavioral therapy; M, motivational approach; DC, drug counseling; SMD, standard mean deviation; CI, confidence interval.

In comparison with active control, CM+DC, there was no significant difference in cannabis-related problems at post-intervention (SMD=-0.41, CI=-0.87; 0.06, C = 1), the 2-month (SMD=-0.30, CI=-0.77; 0.18, C = 1), the 5-month (SMD=-0.36, CI=-0.82; 0.10, C = 1), the 8-month (SMD=-0.46, CI=-0.98; 0.06, C = 1), and the 11-month follow-up (SMD=-0.14, CI=-0.58; 0.31, C = 1) (**Figure 9**).

The odds of abstinence did not differ significantly between CM+DC and the active control at the 3-month (OR = 1.00, CI = 0.26; 3.80, C = 1), and the 6-month follow-up (OR = 0.40, CI = 0.13; 1.25, C = 1). At post-intervention, the proportion of days abstinent did not differ significantly between the two groups (SMD = 0.37, CI=-0.05; 0.79, C = 1). At the 2-month follow-up, the proportion of days abstinent showed a moderate effect (SMD = 0.57, CI = 0.14, 0.99, C = 1) in favor of the active control. This effect was not maintained at the subsequent follow-ups: 5-month (SMD = 0.31, CI=-0.12; 0.74, C = 1), 8-month (SMD = 0.35, CI=-0.08; 0.78, C = 1), and 11-month follow-up (SMD = 0.33, CI=-0.11; 0.78, C = 1).

In summary, CM+DC, does not appear to provide superior benefits compared with an active control group for cannabis-related problems or the odds of abstinence at any time point between post-intervention and 11-month follow-up. A transient moderate effect was observed for the proportion of days abstinent in the short-term follow-up, favoring CM+C.

#### 3.2.10 Cognitive behavioral therapy plus motivational approach and contingency management

CBT+M+CM has been evaluated in four studies, totaling four types of comparisons and including 383 participants (64–66, 70). The comparison groups comprised: 1) CM compared to CBT, 2) CBT+M+CM compared to DC, and 2) CM compared to case management without components for substance use. Of the included studies, two were rated as having a high risk of bias, and two were rated as having some concerns.

**Figure 10:**
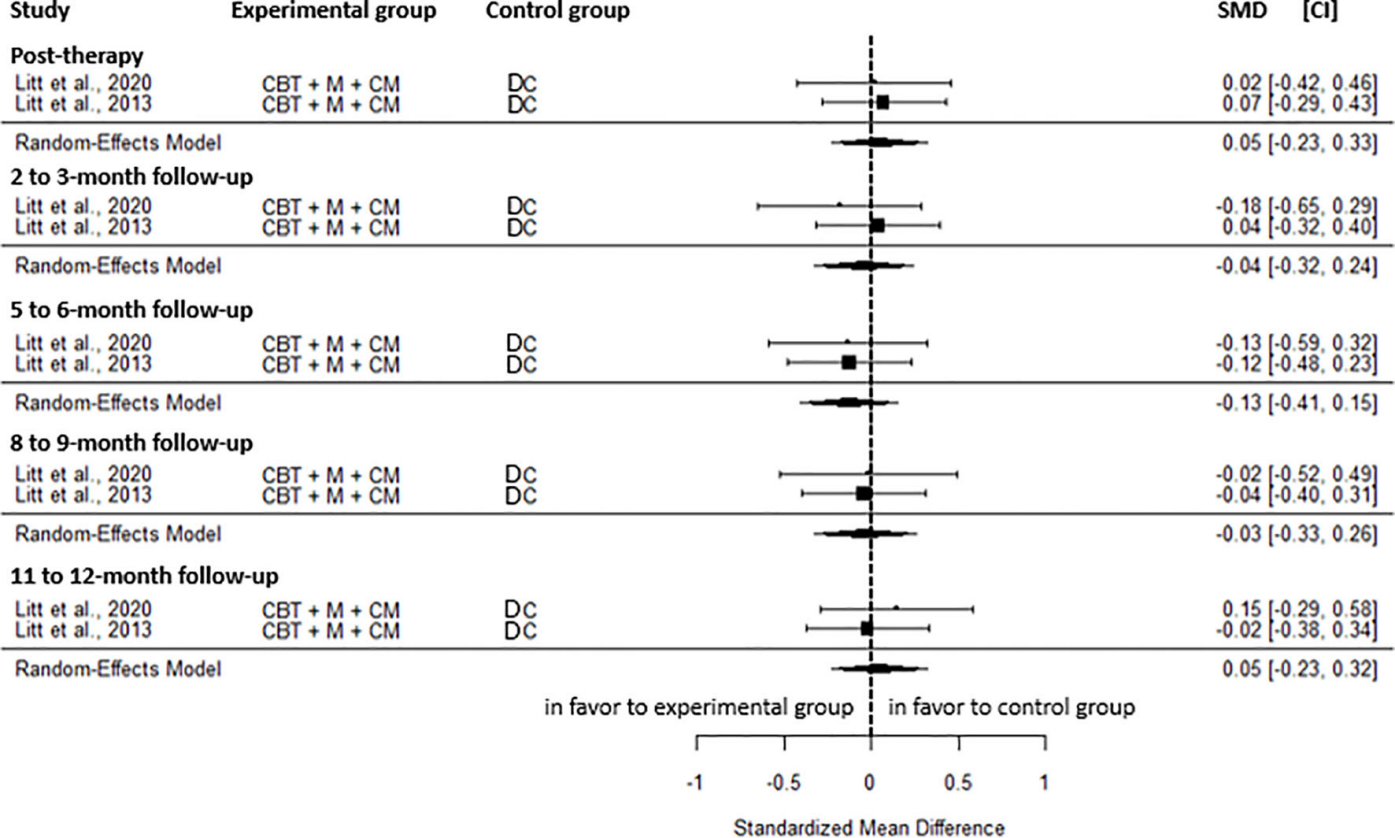
Effect size of cannabis-related problems comparing CBT in addition to motivational and contingency management to an active control group at post-intervention, 2 to 3-month, 5 to 6-month, 8 to 9-month, and 11 to 12-month follow-up. CM, contingency management; CBT, cognitive behavioral therapy; M, motivational approach; DC, drug counseling; SMD, standard mean deviation; CI, confidence interval.

CBT+M+CM was not significantly different to an active control regarding cannabis-related problems at post-intervention (SMD = 0.05, CI=-0.23; 0.33, C = 2), the 2 to 3-month (SMD=-0.04, CI=-0.32; 0.24, C = 2), the 5 to 6-month (SMD=-0.13, CI=-0.41; 0.15, C = 2), the 8 to 9-month (SMD=-0.03, CI=-0.33; 0.26, C = 2), and the 11 to 12-month follow-up (SMD = 0.05, CI=-0.23; 0.32, C = 2) (**Figure 10**). No heterogeneity was observed for these results.

Regarding the odds of abstinence, no significant difference was found between the two groups at post-intervention (OR = 1.54, CI = 0.55; 4.36, C = 1), the 6-month (OR = 1.58, CI = 0.78; 3.22, C = 2, I^2^ = 9.08%), the 9-month (OR = 1.23, CI = 0.53; 2.87, C = 1), and the 12-month follow-up (OR = 1.76, CI = 0.70; 4.44, C = 1). However, weakly increased odds of abstinence in favor of CBT+M+CM were observed at the 3-month follow-up (OR = 2.60, CI = 1.25; 5.41, C = 2). No heterogeneity was observed for this result. Proportion of days of abstinence was not significantly different between groups at post-intervention (SMD = 0.21, CI=-0.12; 0.53, C = 2, I^2^ = 31.05%), the 2 to 3-month (SMD = 0.12, CI=-0.22; 0.46, C = 2, I^2^ = 36.15%), the 5 to 6-month (SMD = 0.22, CI=-0.05; 0.49, C = 2, I^2^ = 0.00%), the 8 to 9-month (SMD=-0.11, CI=-0.39; 0.16, C = 2, I^2^ = 0.00%), and the 11 to 12-month (SMD = 0.01, CI=-0.26; 0.29, C = 2, I^2^ = 0.00%).

Compared to inactive control, CBT+M+CM showed a small effect on readiness to change cannabis (SMD = 0.38, CI = 0.01; 0.75, C = 1) at post-intervention. However, no significant difference was found for self-efficacy (SMD=-0.08; CI=-0.45; 0.29, C = 1) and coping skills (SMD = 0.21, CI=-0.16; 0.58, C = 1). Likely, no significant difference was found for the odds of abstinence at post-intervention (OR = 1.83, CI = 0.63; 5.36, C = 1), the 5-month (OR = 2.09, CI = 0.77; 5.65, C = 1), the 8-month (OR = 1.70, CI = 0.64; 4.51, C = 1), the 11-month (OR = 1.88, CI = 0.71; 4.94, C = 1) and the 14 -month follow-up (OR = 1.59, CI = 0.63; 4.00, C = 1).

In summary, no significant effects were found for cannabis-related problems or the proportion of days abstinent at any time point from post-intervention to 12-month follow-up between CBT+M+CM and the active control group. A transient, weak increase in the odds of abstinence was observed in favor of CBT+M+CM at short-term follow-up. Compared to the inactive control, CBT+M+CM showed a small effect on readiness to change cannabis at post-intervention. No significant difference was found for abstinence at any timepoint from post-intervention to 14-month follow-up.

### 3.3 Publication bias

**Figure 11:**
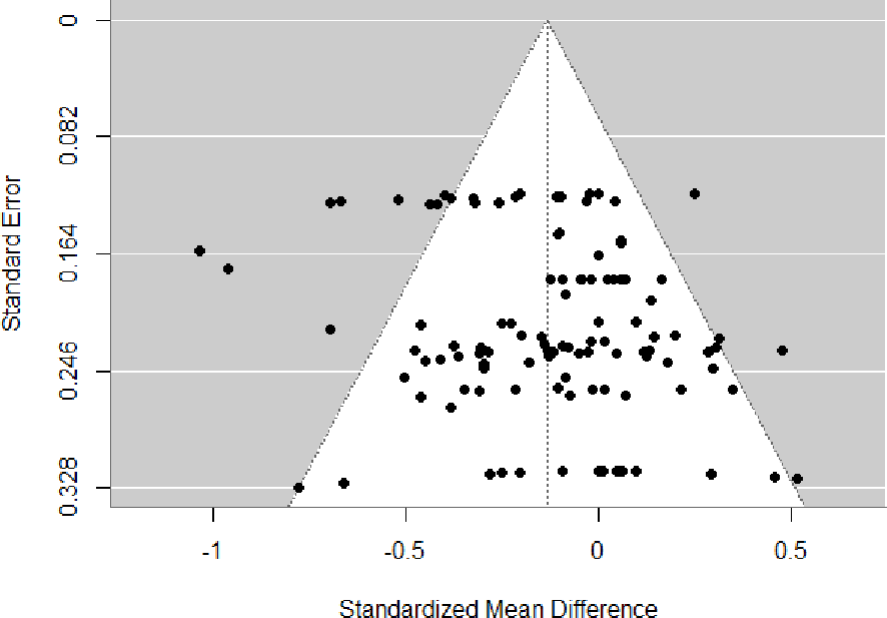
Funnel plot of the meta-analysis of psychotherapy for cannabis use.

An examination of the funnel plot suggested publication bias in the overall database (**Figure 11**). Egger’s test also suggested that there was publication bias (t = 2.39, p = 0.02).

## 4 Discussion

This meta-analysis synthesized the current state of evidence on the efficacy of psychotherapies for individuals using cannabis and assessed the risk of bias ranging from some concerns to high. These meta-analyses provide a valuable overview of the effectiveness of interventions, particularly regarding cannabis-related problems and the severity of cannabis dependence. In summary, regarding the primary outcome of cannabis-related problems, CBT was the only intervention associated with a small effect when compared with an active control group. This result was from on a single study. In addition, based on two studies, CBT+M demonstrated a small effect on cannabis-related problems in the short term only when compared with an inactive control group. Regarding secondary outcomes, when compared with active control groups, only CBT showed small effects on the severity of cannabis dependence, readiness to change, the frequency and quantity of cannabis use at specific time points, while the majority of time points were non-significant. Most of the significant findings were derived from one or two studies. CBT alone, CBT+M, and CBT+M+CM showed transiently slightly higher effects on abstinence at some follow-ups; however, most comparisons across time points were non-significant. Other psychological interventions, including CM, DC, relapse prevention, social support groups, CM+M, CBT+CM, and CM+DC, did not show significant differences in any outcomes compared with active control groups. Comparisons with inactive control groups indicated that CBT alone, as well as CBT+M, produced moderate to large benefits on the severity of cannabis dependence. CBT+M+CM showed a small effect on readiness to change cannabis and coping skills at post-intervention. However, each of the results comes from one study outcomes were measured only at the post-intervention. CBT+M and M+relapse prevention showed large reductions in frequency and quantity of cannabis use at short-term follow-up; M+relapse prevention was also evaluated in only one study. Regarding abstinence, CBT+M showed large effects, whereas CBT+M+CM showed transient weak effects at short-term follow-up. Each of these results came from one or two studies. Neither CM alone nor DC demonstrated significant differences compared to the inactive control.

Most meta-analyses evaluating psychotherapeutic interventions for cannabis use in adults have not specifically reported outcomes related to the severity of cannabis-related problems or CUD (35, 37, 42). Instead, the majority of studies have primarily focused on abstinence or reductions in the frequency and quantity of cannabis use as indicators of treatment effectiveness, while giving less consideration to the negative consequences experienced by users (35, 37, 42). Nevertheless, these outcomes represent a relevant endpoint that warrants consideration. First, from a clinical perspective, the diagnosis of CUD places greater emphasis on the problems and impairments associated with cannabis use rather than on consumption levels alone (75). Although frequency and quantity of use are associated with the severity of cannabis-related problems, individuals with similar levels of consumption may experience highly heterogeneous consequences of varying intensity (76). Clinically, this suggests that some patients may continue to experience significant negative outcomes despite reduced use, underscoring the need for individualized, consequence-focused treatment approaches. Second, with respect to patient-centered treatment goals, individuals seeking psychotherapy do not necessarily aim for complete abstinence but often seek to reduce the harms and difficulties associated with their cannabis use (77). These findings highlight the importance of identifying and targeting therapeutic approaches that better support patients’ goals and effectively minimize cannabis-related consequences (78–80). Indeed, interventions focusing on harm reduction and controlled use may be more appropriate for certain individuals than those exclusively targeting abstinence, particularly when aligned with patient preferences and motivation (81). The low rates of complete abstinence observed across studies further support harm reduction as a more realistic and clinically relevant treatment objective. Current psychotherapies may primarily target reductions in use or abstinence, which could partly explain their limited effectiveness in reducing both consumption and cannabis-related problems. Evaluating psychological interventions that explicitly target the reduction of cannabis-related consequences may therefore represent a promising avenue for future research.

Although this meta-analysis is of interest to clinical practice, these results should be interpreted with caution, as several limitations remain unresolved. Firstly, limitations include those common to all meta-analyses, such as heterogeneity and publication bias. Results often showed small to moderate heterogeneity when reported, suggesting the presence of subgroups of patients who may respond better to the intervention than others. The presence of publication bias suggests the possibility of overestimating results. This reinforces the importance of registering conducted studies. Due to the limited number of studies in each analysis, publication bias was evaluated using the overall pooled dataset. These findings should therefore be interpreted with caution. Secondly, the small number of psychological intervention modalities included in this meta-analysis constitutes an important methodological limitation. Indeed, most significant findings were extracted from one or two combined studies and were observed immediately after the intervention. It limits the robustness of the conclusions that can be drawn. Given the limited number of studies for each outcome, we could not adjust for the inclusion of different outcomes from the same studies using a multilevel approach. However, when tested on all outcomes, the multilevel approach resulted in similar conclusions. Therefore, it was only possible to compare outcomes by control group type (active vs. inactive) rather than by intervention type. This limitation may have introduced heterogeneity in control conditions across studies, potentially biasing the aggregated outcomes and leading to either underestimation or overestimation of the findings. Moreover, it was not possible to account for potential confounding or moderating factors (e.g., sample and treatment characteristics). Indeed, the intensity and duration of interventions may be important considerations, whereas brief interventions (1–2 sessions) may not be efficacious (38). It would also be of interest, once more studies become available, to explore whether a dose–response relationship exists between the number and intensity of sessions and the effectiveness of psychological intervention. Also, sub-analyses based on different sample characteristics (e.g., CUD diagnostic criteria, severity of cannabis-related problems, sex, age) may be relevant. For example, men and women may not respond to psychotherapy in the same way, as reported in a study where men were more likely than women to achieve point-prevalence abstinence during treatment (82). Age at cannabis use initiation also appears to negatively moderate treatment outcomes (83). Furthermore, it is possible that participants with mental health disorders were included in some studies without being systematically reported, which could have led to an underestimation of treatment efficacy, as individuals with comorbid mental health disorders tend to have poorer outcomes (83). Finally, half of the studies had some concerns about risk of bias, and the other half had a high risk of bias. Several factors contributed to the risk of bias, including whether allocation was conducted by an independent person, deviations from the intended interventions, the lack of intention-to-treat analyses, the exclusion of a substantial number of randomized participants from the analyses, and the absence of blinded outcome assessment. This is critical, as an RCT with methodological limitations is insufficient to support evidence-based practice. Therefore, the risk of bias of included studies must be carefully considered when interpreting the efficacy of interventions.

## 5 Conclusion

Given the high prevalence of cannabis use and the wide range of cannabis-related problems affecting users, their families, and society, effective interventions for cannabis use are critically needed. This systematic review and meta-analysis aim to summarize the literature on the efficacy of psychotherapies for cannabis use. Moreover, several gaps persist in the literature on the efficacy of psychosocial treatments to reduce harms related to cannabis use. Currently, the evidence supporting the effectiveness of psychotherapeutic interventions in reducing cannabis-related problems remains very limited. Further studies are needed to strengthen and clarify the quality of the existing evidence. Among the available interventions, CBT appears to be the only approach associated with beneficial effects, although these effects are small. Consequently, the development and evaluation of new treatment modalities targeting cannabis-related problems should be considered.
