# Supplementary material for: The effectiveness of psychological interventions delivered by health professionals for adult cannabis use in community settings: a systematic review and meta-analysis
Source: Front Psychiatry. 2026 Mar 20;17:1792228. doi: 10.3389/fpsyt.2026.1792228 (PMC13048070; doi:10.3389/fpsyt.2026.1792228)
Supplement: Supplementary file 1 [file SupplementaryFile1.docx]

***Supplementary Material***

**The effectiveness of psychological interventions for cannabis use in adults: A systematic review and meta-analysis**

**Table S1.** Summary of measures reported by meta-analyses on the effectiveness of psychological interventions for cannabis use.

**Table S2.** Electronic search strategy for the systematic review conducted.

**Table S3.** Details of the retrieved studies included.

**Table S4.** PRISMA Checklist.

**Table S1. Summary of measures reported by meta-analyses on the effectiveness of psychological interventions for cannabis use.**

| **Studies** | Measures **reported by meta-analyses** |
| --- | --- |
| Halicka et al., 2025 | - Point abstinence and continuous abstinence - Intensity of withdrawal and/or craving - Quantity of use - Frequency of use |
| Tanner-Smith et al., 2021 | - Frequency - Screening for hazardous, harmful and dependent use of cannabis |
| Calomarde-Gómez et al., 2021 | - Abstinence - Quantity of use - Frequency of use - Cannabis use disorder severity |
| Imtiaz et al., 2020 | - Abstinence - Frequency |
| Gates et al., 2016 | - Abstinence - Cannabis-related problems - Frequency |
| White et al., 2015 | - Abstinence - Frequency |
| Magill et al., 2009 | - The outcome indicator for effect size calculation was selected in the following order: (1) biological measures, (2) measures of use frequency, and (3) sample proportions |

**Table S2.** Electronic search strategy used for the present systematic review and meta-analysis

| **Database; Search** | **Search terms** |
| --- | --- |
| **PubMed**;  k=3 091 | (Cannabis[Title/Abstract] OR marijuana[Title/Abstract] OR THC[Title/Abstract] OR tetrahydrocannabinol[Title/Abstract] OR hashish[Title/Abstract]) AND (“Psychosocial treatment”[Title/Abstract] OR “Psychological treatment”[Title/Abstract] OR Psychotherapy[Title/Abstract] OR Therapy[Title/Abstract] OR “Contingency management”[Title/Abstract] OR “case management”[Title/Abstract] OR “Cognitive behavioral therapy”[Title/Abstract] OR CBT[Title/Abstract] OR “Cognitive behaviour therapy”[Title/Abstract] OR “Relapse prevention”[Title/Abstract] OR “Dialectical behavior therapy”[Title/Abstract] OR “Group drug counseling”[Title/Abstract] OR “Motivational interviewing”[Title/Abstract] OR “Motivational enhancement interviewing”[Title/Abstract] OR “Motivational enhancement therapy”[Title/Abstract] OR “12-step facilitation”[Title/Abstract] OR “Social support”[Title/Abstract] OR “Mindfulness-based meditation”[Title/Abstract] OR Psychoeducation[Title/Abstract] OR “Therapeutic community”[Title/Abstract] OR “Skills training”[Title/Abstract] OR “abstinence reinforcement therapy”[Title/Abstract] OR “Brief intervention”[Title/Abstract] OR “Computer-assisted therapy”[Title/Abstract] OR “Family therapy”[Title/Abstract]) |
| **PsycINFO**;  k=2 758 | (Abstract: Cannabis OR Abstract: marijuana OR Abstract: THC OR Abstract: tetrahydrocannabinol OR Abstract: hashish) AND (Abstract: Psychosocial treatment OR Abstract: Psychological treatment OR Abstract: Psychotherapy OR Abstract: Therapy OR Abstract: Contingency management OR Abstract: case management OR Abstract: Cognitive behavioral therapy OR Abstract: CBT OR Abstract: Cognitive behaviour therapy OR Abstract: Relapse prevention OR Abstract: Dialectical behavior therapy OR Abstract: Group drug counseling OR Abstract: Motivational interviewing OR Abstract: Motivational enhancement interviewing OR Abstract: Motivational enhancement therapy OR Abstract: 12-step facilitation OR Abstract: Social support OR Abstract: Mindfulness-based meditation OR Abstract: Psychoeducation OR Abstract: Therapeutic community OR Abstract: Skills training OR Abstract: abstinence reinforcement therapy OR Abstract: Brief intervention OR Abstract: Computer-assisted therapy OR Abstract: Family therapy) |
| **Web of Science**;  k=4 977 | (AB=(Cannabis OR marijuana OR THC OR tetrahydrocannabinol OR hashish)) AND (AB=(Psychosocial treatment OR Psychological treatment OR Psychotherapy OR Therapy OR Contingency management OR case management OR Cognitive behavioral therapy OR CBT OR Cognitive behaviour therapy OR Relapse prevention OR Dialectical behavior therapy OR Group drug counseling OR Motivational interviewing OR Motivational enhancement interviewing/ Motivational enhancement therapy OR 12-step facilitation OR Social support OR Mindfulness-based meditation OR Psychoeducation OR Therapeutic community OR Skills training OR abstinence reinforcement therapy OR Brief intervention OR Computer-assisted therapy OR Family therapy)) |

^Note.^ A search in Google Scholar with the exact keywords and cross-referencing enabled the finding of an additional 63 articles.

**Table S3. Details of the retrieved studies.**

| **Authors, years** | **Design of studies**  **Studies countries** | **Description of sample** | **Comparisons groups** | **Results** | **Risk of bias** |
| --- | --- | --- | --- | --- | --- |
| Babor et al., 2004 | RCT  (no blind assessors)  United States of America | **Criteria related to cannabis**  Diagnosis of current marijuana dependence, and used marijuana on at least 40 of the 90 days prior to the study  **Criteria related to other substances**  Excluded diagnosis of dependence on another drug or alcohol | **Intervention group**  MET + CBT + case management (133)  9 sessions  **Control group**  Brief MET (128)  2 sessions | ***Cannabis-related problems***  Marijuana Problems Scale  **1-month: SMD=-0.52, CI=-0.77; -0.27**  **6-month: SMD=-0.42, CI=-0.67, -0.17**  ***Severity of medical problem***  Addiction Severity Index medical subscale  1-month: SMD=-0.21, CI=-0.46; 0.03  6-month: SMD=-0.03, CI=-0.28; 0.22  ***Severity of employment problem***  Addiction Severity Index employment subscale  1-month: SMD=-0.10, CI=-0.34; 0.15  6-month: SMD=0.05, CI=-0.20; 0.30  ***Severity of psychiatric problem***  Addiction Severity Index psychiatric subscale  1-month: SMD=-0.11, CI=-0.35, 0.14  6-month: SMD=-0.26, CI=-0.51; 0.00  ***Depression***  Beck Depression Inventory  **1-month: SMD=-0.32, CI-0.57; -0.08**  **6-month: SMD=-0.32, CI-0.57; -0.07**  ***Anxiety***  State–Trait Anxiety Inventory state portion  **1-month: SMD=-0.38, CI=-0.63; -0.14**  **6-month: SMD=-0.44, CI=-0.69; -0.18**  ***Dependence symptoms***  Structured Clinical Interview for DSM-IV  **1-month: SMD=-0.53, CI=-0.78, -0.29**  **6-month: SMD=-0.36, CI=-0.62, -0.11**  ***Quantity***  TLFB  **1-month: SMD=-0.30, CI=-0.54; -0.06**  6-month: SMD=-0.05, CI=-0.30, 0.20  ***Frequency***  TLFB  **1-month: SMD=-0.52, CI=-0.77; -0.28**  **6-month: SMD=-0.43, CI=-0.68; -0.17**  12-month: SMD=-0.22, CI=-0.47; 0.03  ***Abstinence***  TLFB  **1-month: OR=3.03, CI=1.45; 6.33**  6-month: OR=1.70, CI=0.80; 3.63  **12-month: OR=2.03, CI=1.03; 4.01** | 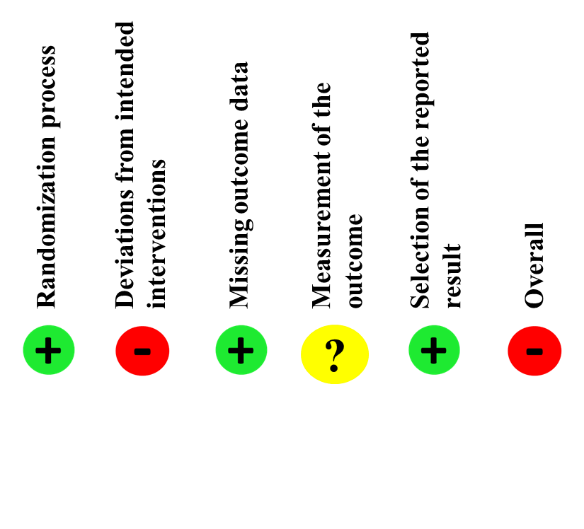 |
|  |  |  | **Intervention group**  MET + CBT + case management (133)  9 sessions  **Control group**  delayed treatment (137) | \| ***Cannabis-related problems***  Marijuana Problems Scale  **1-month: SMD=-0.40, CI=-0.64; -0.16**  ***Severity of medical problem***  Addiction Severity Index medical subscale  **1-month: SMD=0.25, CI=0.01; 0.49**  ***Severity of employment problem***  Addiction Severity Index employment subscale  1-month: SMD=0.00, CI-0.24; 0.24  ***Severity of psychiatric problem***  Addiction Severity Index psychiatric subscale  1-month: SMD=0.00, -0.24; 0.24  **Depression**  Beck Depression Inventory  1-month: SMD=-0.02, CI=-0.26; 0.22  **Anxiety**  State–Trait Anxiety Inventory state portion  1-month: SMD=-0.20, CI=-0.44; 0,04  ***Dependence symptoms***  Structured Clinical Interview for DSM-IV  **1-month: SMD=-0.88, CI=-1.13; -0.63**  ***Abstinence***  TLFB  **1-month: OR=7.76, CI=2.91; 20.72**  ***Quantity***  TLFB  **1-month: SMD=-0.56, CI=-0.80; -0.32**  ***Frequency***  TLFB  **1-month:** **SMD=-1.13, CI=-1.38; -0.87** \| \| --- \| |  |

| **Authors, years** | **Design of studies**  **Studies countries** | **Description of sample** | **Comparisons groups** | **Results** | **Risk of bias** |
| --- | --- | --- | --- | --- | --- |
| Budney et al., 2000 | RCT  (no blind assessors)  United States of America | **Criteria related to cannabis**  diagnosis of current marijuana  dependence, and have used marijuana in the past 30 days.  **Criteria related to other substances**  Excluded current dependence on alcohol or any other drug except nicotine | **Intervention group**  MI + CBT (20)  14 sessions  **Control group**  MI (20)  14 sessions | ***Cannabis-related problems***  Drug Abuse Screening Test adapted for use with marijuana abusers  Post: SMD=0.10, CI-0.52; 0.72  ***Severity of medical problem***  Addiction Severity Index medical subscale  Post: SMD=0.00, CI=-0.62; 0.62  ***Severity of employment problem***  Addiction Severity Index employment subscale  Post: SMD=0.00, CI=-0.62; 0.62  ***Severity of family/social relations problems***  Addiction Severity Index family/social relations subscales  Post: SMD=-0.20, CI=-0.82; 0.42  ***Severity of psychiatric problem***  Addiction Severity Index psychiatric subscale  **Post: SMD=-0.77, CI=-1.42; -0.13**  ***Psychiatric distress***  Brief symptom inventory (global symptom index)  Post: SMD=-0.25, CI=-0.87; 0.37  ***Readiness to change cannabis***  University of Rhode Island Change Assessment  **Post: SMD=0.79, CI=0.15; 1.44** | 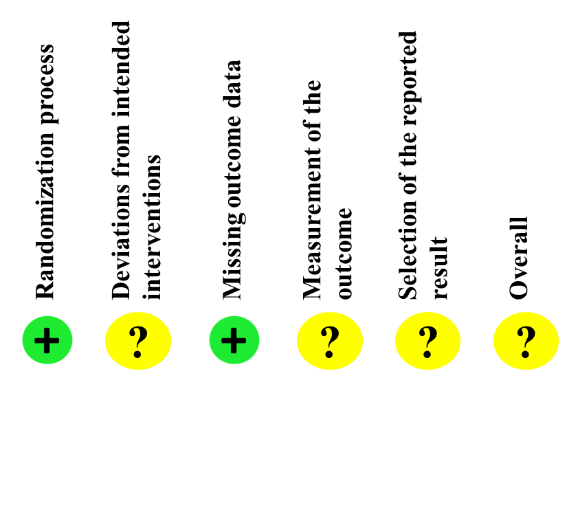 |
|  |  |  | **Intervention group**  MI + CBT + voucher-based incentives (20)  14 sessions  **Control group**  MI + CBT (20)  14 sessions | ***Cannabis-related problems***  Drug Abuse Screening Test adapted for use with marijuana abusers  Post: SMD=0.46, CI=-0.17; 1.09  ***Severity of medical problem***  Addiction Severity Index medical subscale  Post: SMD=0.06, CI=-0.56; 0.68  ***Severity of employment problem***  Addiction Severity Index employment subscale  Post: SMD=0.00, CI=-0.62; 0.62  ***Severity of family/social relations problems***  Addiction Severity Index family/social relations subscales  Post: SMD=-0.09, CI=-0.71; 0.53  ***Severity of psychiatric problem***  Addiction Severity Index  Post: SMD=0.05, CI=-0.57; 0.67  ***Psychiatric distress***  brief symptom inventory (global symptom index)  Post: SMD=0.29, CI=-0.33; 0.92  ***Readiness to change cannabis***  University of Rhode Island Change Assessment  Post: SMD=-0.04, CI=-0.66; 0.58 |  |
|  |  |  | **Intervention group**  MI + CBT + voucher-based incentives (20)  14 sessions  **Control group**  MI (20)  14 sessions | ***Cannabis-related problems***  Drug Abuse Screening Test adapted for use with marijuana abusers  Post: SMD=0.52, CI=-0.11; 1.15  ***Severity of medical problem***  Addiction Severity Index medical subscale  Post: SMD=0.06, CI=-0.56; 0.68  ***Severity of employment problem***  Addiction Severity Index employment subscale  Post: SMD=0.00, CI=-0.62;0.62  ***Severity of family/social relations problems***  Addiction Severity Index family/social relations subscales  Post: SMD=-0.28, CI=-0.90; 0.34  ***Severity of psychiatric problem***  Addiction Severity Index psychiatric subscale  **Post: SMD=-0.66, CI=-1.29; -0.02**  ***Psychiatric distress***  brief symptom inventory (global symptom index)  Post: SMD=0.01, CI=-0.61; 0.63  ***Readiness to change cannabis***  University of Rhode Island Change Assessment  **Post: SMD=0.69, CI=0.05; 1.33** |  |

| **Authors, years** | **Design of studies**  **Studies countries** | **Description of sample** | **Comparisons groups** | **Results** | **Risk of bias** |
| --- | --- | --- | --- | --- | --- |
| Budney et al., 2006 | RCT  (no blind assessors)  United States of America | **Criteria related to cannabis**  Diagnosis of current cannabis dependence, and have used marijuana in the past 30 days  **Criteria related to other substances**  Excluded current dependence on alcohol or any other drug except nicotine | **Intervention group**  Abstinence-based vouchers (30)  14 sessions  **Control group**  CBT (30)  14 sessions | ***Cannabis-related problems***  Marijuana Problem Scale  Post: SMD=-0.21, CI=-0.72; 0.29  ***Quantity***  TLFB  Post: SMD=0.47, CI=-0.04; 0.98  ***Frequency***  TLFB  Post: SMD=0.28, CI=-0.23; 0.79  3-month: SMD=0.31, CI=-0.20; 0.82  **6-month: SMD=0.32, CI=-0.19; 0.83**  9-month: SMD=0.18, CI=-0.33; 0.69  12-month: SMD=-0.01, CI=-0.52; 0.49  ***Abstinence***  Urine test  Post: OR=1.56, CI=0.53; 4.53  3-month: OR=0.80, CI=0.22; 2.07  6-month: OR=1.98, CI=0.51; 7.63  9-month: OR=1.30, CI=0.31; 5.40  12-month: OR=0.66, CI=0.18; 2.36 | 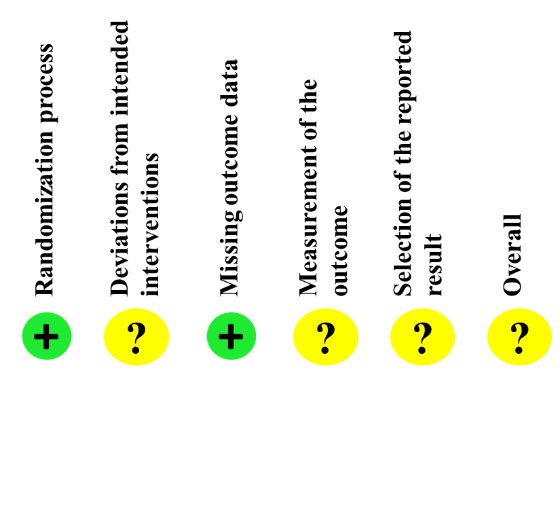 |
|  |  |  | **Intervention group**  CBT + Abstinence-based vouchers (30)  14 sessions  **Control group**  CBT (30)  14 sessions | ***Cannabis-related problems***  Marijuana Problem Scale  Post: SMD=-0.31, CI=-0.82; 0.20  ***Frequency***  TLFB  Post: SMD=0.12, CI=-0.39; 0.63  3-month: SMD=-0.07, CI=-0.58; 0.43  6-month: SMD=-0.16, CI=-0.67; 0.35  9-month: SMD=-0.36, CI=-0.87; 0.15  12-month: SMD=-0.39, CI=-0.90; 0.12  ***Quantity***  TLFB  Post: SMD=0.45, CI=-0.06; 0.96  ***Abstinence***  Urine test  Post: OR=1.78, CI=0.62; 5.17  3-month: OR=3.06, CI=0.97; 9.66  6-month: OR=3.25, CI=0.89; 11.90  9-month: OR=3.25, CI=0.89; 11.90  12-month: OR=1.90, CI=0.62; 5.86 |  |
|  |  |  | **Intervention group**  CBT + Abstinence-based vouchers (30)  14 sessions  **Control group**  Abstinence-based vouchers (30)  14 sessions | ***Cannabis-related problems***  Marijuana Problem Scale  Post: SMD=-0.10, CI=-0.61; 0.40  ***Quantity***  TLFB  Post: SMD=0.04, CI=-0.47; 0.54  ***Frequency***  TLFB  Post: SMD=-0.17, CI=-0.67; 0.34  3-month: SMD=-0.40, CI=-0.91; 0.11  6-month: SMD=-0.49, CI=-1.01; 0.02  **9-month: SMD=-0.56, CI=-1.08; -0.05**  12-month: SMD=-0.40, CI=-0.91; 0.11  ***Abstinence***  Urine test  Post: OR=1.15, CI=0.41; 3.20  **3-month: OR=3.82, CI=1.15; 12.71**  6-month: OR=1.64, CI=0.53; 5.12  9-month: OR=2.50, CI=0.74; 8.50  12-month: OR=2.89, CI=0.86; 9.74 |  |
|  |  |  | **Intervention group**  CBT (30)  14 sessions  **Control group**  Abstinence-based vouchers (30)  14 sessions | ***Cannabis-related problems***  Marijuana Problem Scale  Post: SMD=0.21, CI=-0.29; 0.72  ***Quantity***  TLFB  Post: SMD=-0.47, CI=-0.98; 0.04  ***Frequency***  TLFB  Post: SMD=-0.28, CI=-0.79; 0.,23  3-month: SMD=-0.31, CI=-0.82; 0.20  6-month: SMD=-0.32, CI=-0.83; 0.19  9-month: SMD=-0.18, CI=-0.69; 0.33  12-month: SMD=0.01, CI=-0.49; 0.52  ***Abstinence***  Urine test  Post: OR=0.64, CI=0.22; 1.87  3-month: OR=1.25, CI=0.34; 4.64  6-month: OR=0.51, CI=0.13; 1.95  9-month: OR=0.77, CI=0.19; 3.20  12-month: OR=1.52, CI=0.42, CI=5.47 |  |

| **Authors, years** | **Design of studies**  **Studies countries** | **Description of sample** | **Comparisons groups** | | **Results** | **Risk of bias** |
| --- | --- | --- | --- | --- | --- | --- |
| Carroll et al., 2006 | RT  (no blind assessors)  United States of America | **Criteria related to cannabis**  Current marijuana dependence or submit  a marijuana-positive urine specimen at baseline  **Criteria related to other substances**  Excluded severe substance  dependence that required inpatient treatment and detoxification and current physical dependence on alcohol or opioids | **Intervention group**  Individual drug counseling + CM (24)  8 sessions  **Control group**  Individual drug counseling (30)  8 sessions | | ***Abstinence***  TLFB  3-month: OR=2.38, CI=0.55; 10.20  6-month: OR=1.00, CI=0.32; 3.12  Urine test  3-month: OR=1.42, CI=0.37; 5.47  6-month: OR=2.10, CI=0.70; 6.26 | 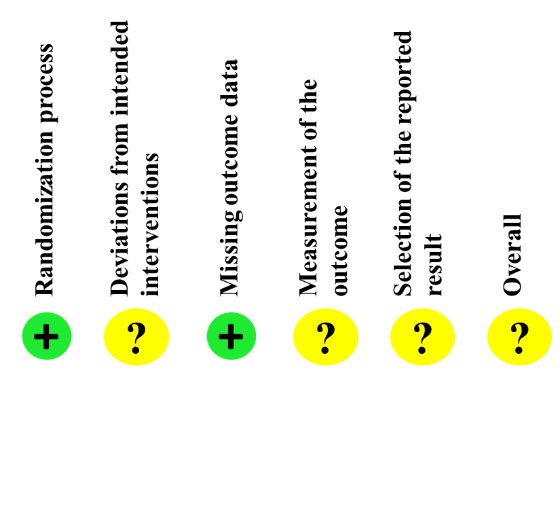 |
|  |  |  | **Intervention group** MET + CBT (27)  8 sessions  **Control group**  Individual drug counseling + CM (24)  8 sessions | | ***Abstinence***  TLFB  3-month: OR=1.00, CI=0.26; 3.80  6-month: OR=2.50, CI=0.80; 7.81  Urine test  3-month: OR=0.80, CI=0.20; 3.13  6-month: OR=0.77, CI=0.25; 2.33 |  |
|  |  |  | **Intervention group** MET + CBT (27)  8 sessions  **Control group**  Individual drug counseling (30)  8 sessions | | ***Abstinence***  TLFB  3-month: OR=2.38, CI=0.58; 9.72  6-month: OR=2.50, CI=0.85; 7.31  Urine test  3-month: OR=1.13, CI=0.30; 4.27  6-month: OR=1.62, CI=0.57; 4.62 |  |
|  |  |  | **Intervention group** MET + CBT + CM (27)  8 sessions  **Control group**  MET + CBT (27)  8 sessions | | ***Abstinence***  TLFB  3-month: OR=1.38, CI=0.40; 4.80  6-month: OR=0.40, CI=0.13; 1.21  Urine test  3-month: OR=2.08, CI=-0.59; 7.38  6-month: OR=0.74, CI=0.25; 2.17 |  |
|  |  |  | **Intervention group** MET + CBT + CM (27)  8 sessions  **Control group**  Individual drug counseling + CM (24)  8 sessions | | ***Abstinence***  TLFB  3-month: OR=1.38, CI=0.38; 5.07  6-month: OR=1.00, CI=0.31; 3.21  Urine test  3-month: OR=1.67, CI=0.46; 6.06  6-month: OR=0.57, CI=0.19; 1.74 |  |
|  |  |  | **Intervention group** MET + CBT + CM (27)  8 sessions  **Control group**  Individual drug counseling (30)  8 sessions | | ***Abstinence***  TLFB  3-month: OR=3.29, CI=0.83; 12.98  6-month: OR=1.00, CI=0.33; 3.01  Urine test  3-month: OR=2.36, CI=0.67; 8.27  6-month: OR=1.20, CI=0.42; 3.44 |  |
| **Authors, years** | **Design of studies**  **Studies countries** | **Description of sample** | **Comparisons groups** | **Results** | | **Risk of bias** |
| Copeland et al., 2001 | RCT  (blind assessors)  Australia | **Criteria related to cannabis**  Must have expressed a  desire to cease cannabis use  **Criteria related to other substances**  More than weekly use of drugs other than cannabis, nicotine, or alcohol in the past six months were excluded. | **Intervention group** CBT + MI + relapse prevention (78)  6 sessions  **Control group**  Delayed Treatment (69) | | ***Cannabis-related problems***  Cannabis Problems Questionnaire  **4-month: SMD=-0.96, CI=-1.30; -0.62**  ***Psychological distress***  Global severity Index subscale of SCL -90-R  4-month: SMD=0.00, CI=-0.32; 0.32  ***Severity of cannabis dependence***  Severity of Dependence Scale  **4-month: SMD=-0.88, CI=-1.22; -0.55**  ***Abstinence***  TLFB  4-month: OR=0.99, CI=0.50; 1.93  ***Quantity***  TLFB  **4-month: SMD=-0.52, CI=-0.85; -0.20** | 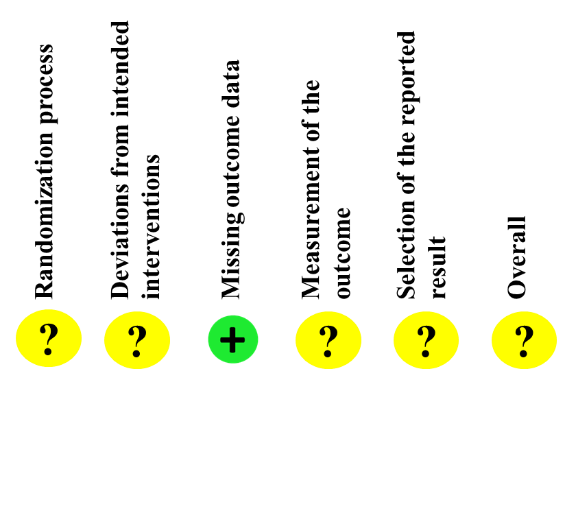 |
| Hoch et al., 2012 | RCT  (blind assessors)  Germany | **Criteria related to cannabis**  Current regular cannabis use defined as use of any cannabis product at least twice a week in the past month, meeting DSM-IV  criteria for lifetime cannabis abuse or dependence  **Criteria related to other substances**  Excluded current DSM-IV-dependence on  alcohol or any other illicit drug with symptoms of withdrawal, tolerance  or craving | **Intervention group** CBT + MET + problem-solving training (79)  10 sessions  **Control group**  Delayed Treatment (31) | | ***Disability days***  CIDI disability days index  **Post: SMD=-0.46, CI=-0.88; -0.04**  ***Severity of medical problems***  Addiction Severity Index medical subscale  Post: SMD=-0.22, CI=-0.64, 0.19  ***Severity of employment problem***  Addiction Severity Index employment subscale  Post: SMD=0.00, CI=-0.42; 0.42  ***Satisfaction***  Addiction Severity Index satisfaction subscale  Post: SMD=0.10, CI=-0.32; 0.51  ***Severity of family/social relations problems***  Addiction Severity Index family/social relations subscales  Post: SMD=-0.25, CI=-0.67; 0.17  ***Psychiatric symptoms***  Brief Symptom Inventory  **Post: SMD=-0.69, CI=-1.12; -0.27**  ***Quantity***  TLFB  **Post: SMD=-0.71, CI=-1.14; -0.29**  ***Abstinence***  TLFB  **Post: OR=6.58, CI=2.11; 20.56** | 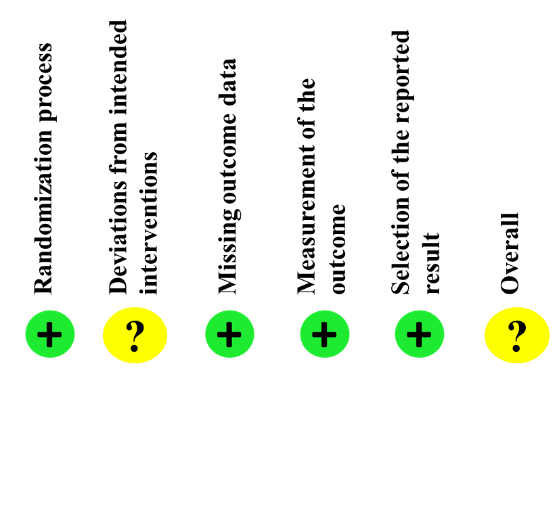 |

| **Authors, years** | **Design of studies**  **Studies countries** | **Description of sample** | **Comparisons groups** | **Results** | **Risk of bias** |
| --- | --- | --- | --- | --- | --- |
| Hoch et al., 2014 | RCT  (blind assessors)  Germany | **Criteria related to cannabis**  Use of any cannabis product at least twice per week over the course of the past 12 months  **Criteria related to other substances**  Excluded ICD-10 dependence in the previous 12 months on alcohol or any other illicit drug (apart from cannabis) with symptoms of withdrawal, tolerance or craving, current use of opiate substitution or sedative-hypnotic medication. | **Intervention group** CBT + MET + problem-solving training (166)  10 sessions  **Control group**  Delayed Treatment (106) | ***Cannabis-related problems***  Cannabis Problems Questionnaire  **Post: SMD=-0.67, CI=-0.92; -0.42**  ***Severity of cannabis use problems***  Cannabis Use Problems Identification Test.  **Post: SMD=-0.70, CI=-0.95; -0.44**  ***Severity of cannabis dependence***  Severity of Dependence Scale  **Post: SMD=-0.55, CI=-0.80; -0.30**  ***Dependence cannabis symptoms***  ICD-10 criteria of dependence  **Post: SMD=-0.83, CI=-1.08; -0.57**  ***Quantity***  TLFB  **Post: SMD=-0.72, CI=-0.97; -0.47**  ***Abstinence***  TLFB  **Post: OR=4.07, CI=2.43; 7.08** | 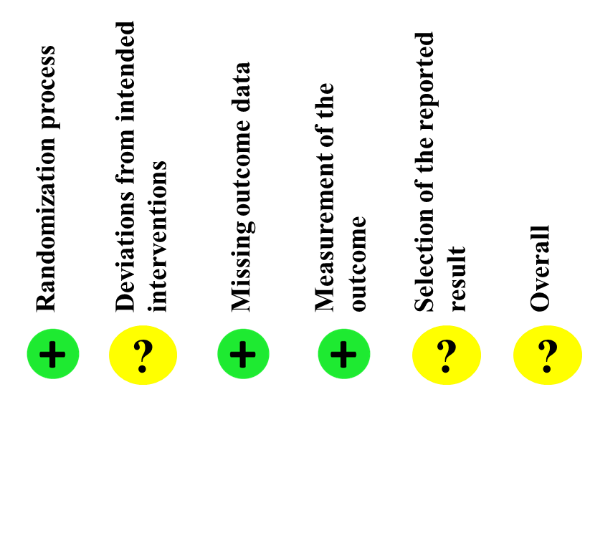 |
| Jungerman et al., 2007 | RCT  (no blind assessors)  Brazil | **Criteria related to cannabis**  Smoked marijuana at least 40 times in the 90 days prior to interview (i.e., 3 times per week).  **Criteria related to other substances**  Excluded criteria for dependence on any other drug (or alcohol), | **Intervention group** MI + relapse prevention (4 sessions in 1-month) (56)  4 sessions  **Control group**  Delayed Treatment (52) | ***Cannabis-related problems***  Marijuana Problems Scale  3-month: SMD=-0.09, CI=-0.46; 0.29  ***Severity of cannabis dependence***  Substance-dependence subscale from the DSM-III-R checklist  3-month: SMD=-0.37, CI=-0.75; 0.01  ***Quantity***  TLFB  **3-month: SMD=-0.59, CI=-0.98; -0.21**  ***Frequency***  TLFB  **3-month: SMD=-0.92, CI=-1.32; -0.53**  ***Abstinence***  TLFB  3-month: OR=0,45, CI=0.04; 5.17 | 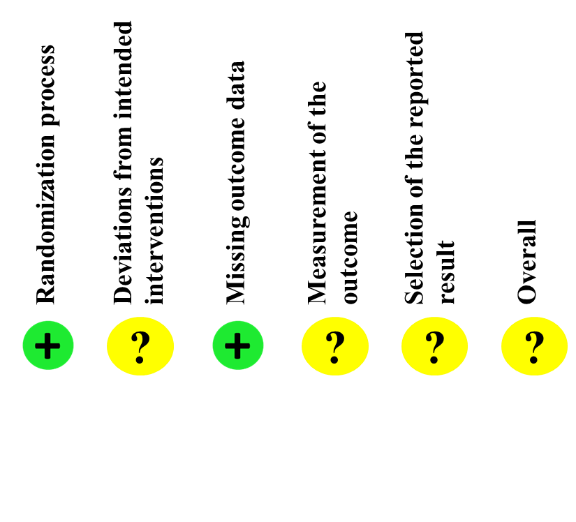 |
|  |  |  | **Intervention group** MI + relapse prevention (4 sessions in 3-month) (56)  4 sessions  **Control group**  Delayed Treatment (52) | ***Cannabis-related problems***  Marijuana Problems Scale  1-month: SMD=0.14, CI=-0.25; 0.52  ***Severity of cannabis dependence***  Substance-dependence subscale from the DSM-III-R checklist  1-month: SMD=-0.10, CI=-0.49; 0.28  ***Quantity***  TLFB  **1-month: SMD=-0.61, CI=-1.01; -0.22**  ***Frequency***  TLFB  **1-month: SMD=-0.68, CI=-1.07; -0.28**  ***Abstinence***  TLFB  1-month: OR=1.53, CI=0.25; 9.56 |  |
| **Authors, years** | **Design of studies**  **Studies countries** | **Description of sample** | **Comparisons groups** | **Results** | **Risk of bias** |
| Litt et al., 2008 | RCT  (no blind assessors)  United States of America | **Criteria related to cannabis**  Diagnosis of current cannabis dependence  **Criteria related to other substances**  Excluded for current alcohol or drug dependence | **Intervention group**  CM (50)  9 sessions  **Control group**  Case Management (no skills relevant to managing substance use were taught) (54)  9 sessions | ***Readiness to change cannabis***  Readiness to Change Questionnaire  Post: SMD=0.35, CI=-0.04; 0.74  ***Self-efficacy***  20-item modification of a smoking cessation self-efficacy questionnaire  Post: SMD=0.21, CI=-0.18; 0.59  ***Coping Skills***  Coping Strategies Scale  Post: SMD=0.02, CI=-0.37; 0.40  ***Abstinence***  TLFB  Post: OR=2.26, CI=0.77; 6.65  5-month: OR=1.53, CI=0.53; 4.36  8-month: OR=0.78, CI=0.25; 2.45  11-month: OR=0.79, CI=0.25; 2.46  14-month: OR=0.60, CI=0.20; 1.80 | 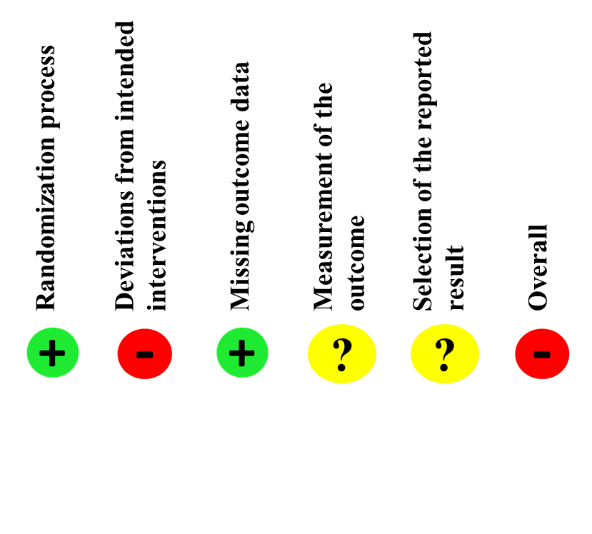 |
|  |  |  | **Intervention group** MET + CBT (55)  9 sessions  **Control group**  Case Management (no skills relevant to managing substance use were taught) (54)  9 sessions | ***Readiness to change cannabis***  Readiness to Change Questionnaire  Post: SMD=0.30, CI=-0.08; 0.68  ***Self-efficacy***  20-item modification of a smoking cessation self-efficacy questionnaire  Post: SMD=0.23, CI=-0.15; 0.60  ***Coping Skills***  Coping Strategies Scale  Post: SMD=0.08, CI=-0.30; 0.46  ***Abstinence***  TLFB  Post: OR=1.17, CI=0.37; 3.73  5-month: OR=1.87, CI=0.68; 5.20  8-month: OR=1.28, CI=0.46; 3.54  11-month: OR=1.00, CI=0.34; 2.90  14-month: OR=1.28, CI=0.46; 3.54 |  |
|  |  |  | **Intervention group** MET + CBT (55)  9 sessions  **Control group**  CM (50)  9 sessions | ***Readiness to change cannabis***  Readiness to Change Questionnaire  Post: SMD=-0.05, CI=-0.43; 0.33  ***Self-efficacy***  20-item modification of a smoking cessation self-efficacy questionnaire  Post: SMD=0.03, CI=-0.35; 0.41  ***Coping Skills***  Coping Strategies Scale  Post: SMD=0.07, CI=-0.32; 0.45  ***Abstinence***  TLFB  Post: OR=0.52, CI=0.18; 1.46  5-month: OR=1.24, CI=0.47; 3.26  8-month: OR=1.63, CI=0.54; 4.87  11-month: OR=1.27, CI=0.41; 3.98  14-month: OR=1.79, CI=0.60; 5.40 |  |
|  |  |  | **Intervention group** MET + CBT + CM (59)  9 sessions  **Control group**  Case Management (no skills relevant to managing substance use were taught) (54)  9 sessions | ***Readiness to change cannabis***  Readiness to Change Questionnaire  **Post: SMD=0.38, CI=0.01; 0.75**  ***Self-efficacy***  20-item modification of a smoking cessation self-efficacy questionnaire  Post: SMD=-0.08; CI=-0.45; 0.29  ***Coping Skills***  Coping Strategies Scale  Post: SMD=0.21, CI=-0.16; 0.58  ***Abstinence***  TLFB  Post: OR=1.83, CI=0.63; 5.36  5-month: OR=2.09, CI=0.77; 5.65  8-month: OR=1.70, CI=0.64; 4.51  11-month: OR=1.88, CI=0.71; 4.94  14-month: OR=1.59, CI=0.63; 4.00 |  |
|  |  |  | **Intervention group** MET + CBT + CM (59)  9 sessions  **Control group**  CM (50)  9 sessions | ***Readiness to change cannabis***  Readiness to Change Questionnaire  Post: SMD=-0.03, CI=-0.40; 0.35  ***Self-efficacy***  20-item modification of a smoking cessation self-efficacy questionnaire  Post: SMD=-0.31, CI=-0.69; 0.07  ***Coping Skills***  Coping Strategies Scale  Post: SMD=0.19, CI=-0.18; 0.57  ***Abstinence***  TLFB  Post: OR=0.81, CI=0.32; 2.07  5-month: OR=1.38, CI=0.54; 3.54  8-month: OR=2.17, CI=0.75; 6.23  11-month: OR=2.39, CI=0.84; 6.82  14-month: OR=2.65, CI=0.92; 7.60 |  |
|  |  |  | **Intervention group** MET + CBT + CM (59)  9 sessions  **Control group**  MET + CBT (55)  9 sessions | ***Readiness to change cannabis***  Readiness to Change Questionnaire  Post: SMD=0.03, CI=-0.34; 0.40  ***Self-efficacy***  20-item modification of a smoking cessation self-efficacy questionnaire  Post: SMD=-0.32, CI=-0.69; 0.05  ***Coping Skills***  Coping Strategies Scale  Post: SMD=0.13, CI=-0.24; 0.50  ***Abstinence***  TLFB  Post: OR=1.57, CI=0.56; 4.40  5-month: OR=1.11, CI=0.46; 2.68  8-month: OR=1.33, CI=0.53; 3.36  11-month: OR=1.88, CI=0.71; 4.94  14-month: OR=1.48, CI=0.58; 3.73 |  |
| **Authors, years** | **Design of studies**  **Studies countries** | **Description of sample** | **Comparisons groups** | **Results** | **Risk of bias** |
| Litt et al., 2013 | RCT  (no blind assessors)  United States of America | **Criteria related to cannabis**  Diagnosis of marijuana dependence or  abuse  **Criteria related to other substances**  Excluded current dependence on drugs other than nicotine and marijuana | **Intervention group**  MET + CBT + CM (60)  9 sessions  **Control group**  Case Management (61)  9 sessions | ***Cannabis-related problems***  Marijuana Problems Scale  Post: SMD=0.07, CI=-0.29; 0.43  3-month: SMD=0.04, CI=-0.32; 0.40  6-month: SMD=-0.12, CI=-0.48, 0.23  9-month: SMD=-0.04, CI=-0.40; 0.31  12-month: SMD=-0.02, CI=-0.38; 0.34  ***Proportion days abstinent***  TLFB  **Post: SMD=0.36, CI=0.00; 0.72**  3-month: SMD=0.28, CI=-0.08; 0.64  6-month: SMD=0.32, CI=-0.04; 0.68  9-month: SMD=0.00, CI=-0.36; 0.36  12-month: SMD=0.11, CI=-0.25; 0.47  ***Abstinence***  TLFB  Post: OR=1.54, CI=0.55; 4.36  3-month: OR=2.36, CI=0.99; 5.64  6-month: OR=2.11, CI=0.90; 4.93  9-month: OR=1.23, CI=0.53; 2.87  12-month: OR=1.76, CI=0.70; 4.44 | 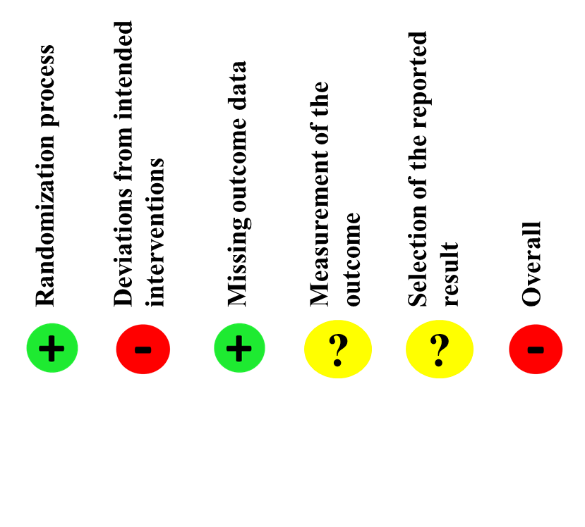 |

| **Authors, years** | **Design of studies**  **Studies countries** | **Description of sample** | **Comparisons groups** | **Results** | **Risk of bias** |
| --- | --- | --- | --- | --- | --- |
| Litt et al., 2020 | RCT  (no blind assessors)  United States of America | **Criteria related to cannabis**  Diagnosis of cannabis  dependence  **Criteria related to other substances**  Could meet criteria for dependence on other substances, but must have reported that marijuana was their primary  substance of abuse | **Intervention group**  MET + CBT (44)  9 sessions  **Control group**  Individualized Assessment and Treatment Program (46)  9 sessions | ***Cannabis- related problems***  Marijuana Problems Scale  Post: SMD=0.31, CI=-0.15; 0.76  2-month: SMD=0.30, CI=-0.18; 0.78  5-month: SMD=0.28, CI=-0.17; 0.74  8-month: SMD=0.35, CI=-0.16; 0.86  **11-month: SMD=0.47, CI=0.02; 0.93**  ***Abstinence***  TLFB  **Post: SMD=-0.42, CI=-0.84; 0.00**  **2-month: SMD=-0.61, CI=-1.04; -0.19**  5-month: SMD=-0.38, CI=-0.81; 0.05  **8-month: SMD=-0.57, CI=-1.01; -0.13**  11-month: SMD=-0.44, CI=-0.89; 0.01 | 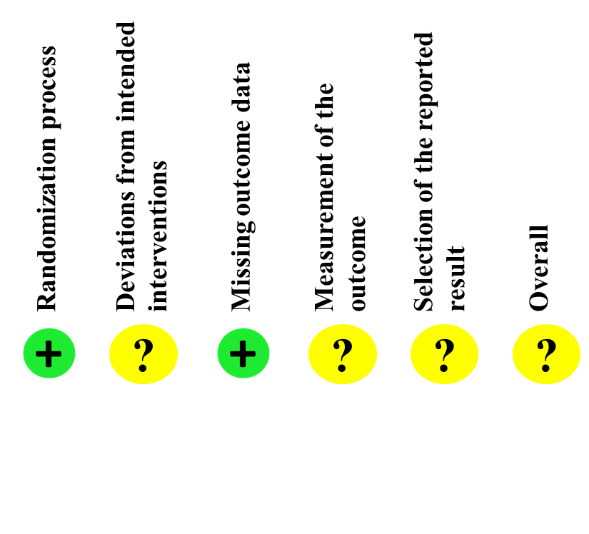 |
|  |  |  | **Intervention group**  MET + CBT + CM (46)  9 sessions  **Control group**  Individualized Assessment and Treatment Program (46)  9 sessions | ***Cannabis- related problems***  Marijuana Problems Scale  Post: SMD=0.02, CI=-0.42; 0.46  2-month: SMD=-0.18, CI=-0.65; 0.29  5-month: SMD=-0.13, CI=-0.59; 0.32  8-month: SMD=-0.02, CI=-0.52; 0.49  11-month: SMD=0.15, CI=-0.29; 0.58  ***Abstinence***  TLFB  Post: SMD=0.02, CI=-0.38; 0.43  2-month: SMD=-0.07, CI=-0.48; 0.34  5-month: SMD=-0.10, CI=-0.32; 0.51  8-month: SMD=-0.28, CI=-0.71; 0.15  11-month: SMD=-0.14, CI=-0.57; 0.30 |  |
|  |  |  | **Intervention group**  MET + CBT + CM (46)  9 sessions  **Control group**  MET + CBT (44)  9 sessions | ***Cannabis- related problems***  Marijuana Problems Scale  Post: SMD=-0.31, CI=-0.76; 0.15  **2-month: SMD=-0.50, CI=-0.99; -0.01**  5-month: SMD=0.45, CI=-0.91; 0.02  8-month: SMD=-0.38, CI=-0.91; 0.15  11-month: SMD=-0.38, CI=-0.82, 0.07  ***Abstinence***  TLFB  **Post: SMD=0.46, CI=0.04; 0.88**  **2-month: SMD=0.55, CI=0.12; 0.98**  **5-month: SMD=0.50, CI=0.06; 0.93**  8-month: SMD=0.28, CI=-0.16; 0.71  **11-month: SMD=0.31, CI=0.14; 0.75** |  |
|  |  |  | **Intervention group**  MET + CBT + CM (46)  9 sessions  **Control group**  CM + Individualized Assessment and Treatment Program (46)  9 sessions | ***Cannabis- related problems***  Marijuana Problems Scale  Post: SMD=0.12, CI=-0.34; 0.57  2-month: SMD=-0.13, CI=-0.59; 0.34  5-month: SMD=-0.05, CI=-0.51; 0.41  8-month: SMD=0.07, CI=-0.44; 0.59  11-month: SMD=-0.20, CI=-0.63; 0.23  ***Abstinence***  TLFB  Post: SMD=0.07, CI=-0.34; 0.48  2-month: SMD=-0.02, CI=-0.44; 0.39  5-month: SMD=0.17, CI=-0.25; 0.59  8-month: SMD=-0.07, CI=-0.49; 0.35  11-month: SMD=-0.02, CI=-0.46; 0.41 |  |
|  |  |  | **Intervention group**  MET + CBT (44)  9 sessions  **Control group**  CM + Individualized Assessment and Treatment Program (46)  9 sessions | ***Cannabis- related problems***  Marijuana Problems Scale  Post: SMD=0.12, CI=-0.34; 0.57  2-month: SMD=-0.13, CI=-0.59; 0.34  5-month: SMD=-0.05, CI=-0.51; 0.41  8-month: SMD=0.07, CI=-0.44; 0.59  11-month: SMD=-0.20, CI=-0.63; 0.23  ***Abstinence***  TLFB  Post: SMD=-0.37, CI=-0.79; 0.05  **2-month: SMD=-0.57, CI=-0.99; -0.14**  5-month: SMD=-0.31, CI=-0.74; 0.12  8-month: SMD=-0.35, CI=-0.78; 0.08  11-month: SMD=-0.33, CI=-0.78; 0.11 |  |
|  |  |  | **Intervention group**  CM + Individualized Assessment and Treatment Program (46)  9 sessions  **Control group**  Individualized Assessment and Treatment Program (46)  9 sessions | ***Cannabis- related problems***  Marijuana Problems Scale  Post: SMD=-0.09, CI=-0.54; 0.36  2-month: SMD=-0.03, CI=-0.48; 0.43  5-month: SMD=-0.08, CI=-0.53; 0.37  8-month: SMD=-0.09, CI=-0.58; 0.41  11-month: SMD=0.31, CI=-0.13; 0.75  ***Abstinence***  TLFB  Post: SMD=-0.05, CI=-0.46; 0.36  2-month: SMD=-0.05, CI=-0.45; 0.36  5-month: SMD=-0.07, CI=-0.48; 0.35  8-month: SMD=-0.21, CI=-0.63; 0.21  11-month: SMD=-0.11, CI=-0.55; 0.32 |  |
| **Authors, years** | **Design of studies**  **Studies countries** | **Description of sample** | **Comparisons groups** | **Results** | **Risk of bias** |
| Pèlerin et al., 2025 | RCT  (no blind assessors)  France | **Criteria related to cannabis**  Regular use of cannabis at  least 7 times per week,  dependence or abuse  associated to  Mini-International  Neuropsychiatric  Interview of at least 4 days per week, covering eight days of consumption in the last 2 weeks. Volunteers to stopping cannabis consumption  **Criteria related to other substances**  Excluded substance dependence or abuse. alcohol and other substances | **Intervention group**  Mindfulness-based relapse prevention (16)  8 sessions  **Control group**  TAU (standard outpatient care  provided by a psychiatrist  trained in addictology with experience of cannabis user care.) (16) | ***Withdrawal symptoms***  Cannabis Withdrawal Scale  **Post: SMD=-1.84, CI=-2.73; -0.95**  1-month: SMD=-1.16, CI=-2.36; 0.04  ***Frequency***  TLFB  Post: SMD=0.79, CI=-1.75, -0.02  **1-month: SMD=-1.37, CI=-2.60; -0.13** | 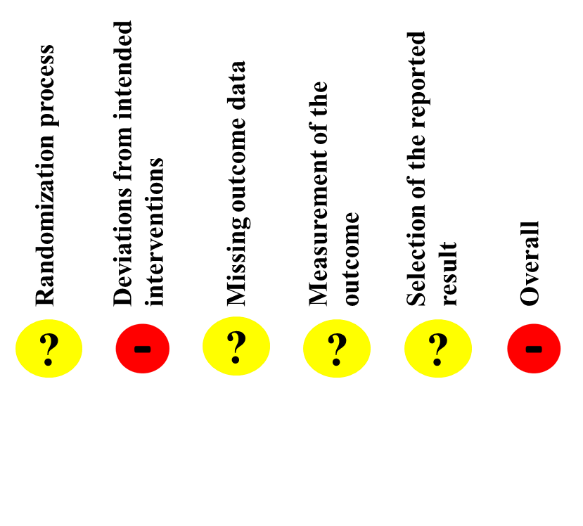 |
| Sinha et al., 2003 | RT  (no blind assessors)  United States of America | **Criteria related to cannabis**  Diagnosis of marijuana abuse or dependence  **Criteria related to other substances**  Excluded currently abusing opiates or cocaine, or whose principal substance of abuse was not marijuana | **Intervention group**  MET + CM (37  3 sessions  **Control group**  MET (28)  3 sessions | ***Severity of cannabis dependence***  Addiction Severity Index  Post: SMD=0.18, CI=-0.32; 0.67  1-month: SMD=0.11, CI=-0.38; 0.60  ***Readiness to change cannabis***  Stages of Change Readiness and Treatment Eagerness Scale  Post: SMD=0.29, CI=-0.21; 0.78  1-month: SMD=0.22, CI=-0.27, 0.71  ***Frequency***  Questionnaire maison (self-report (using a weekly substance use calendar)  **Post: SMD=0.50, CI=0.00;1.00**  1-month: SMD=0.22, CI=-0.27; 0.71 | 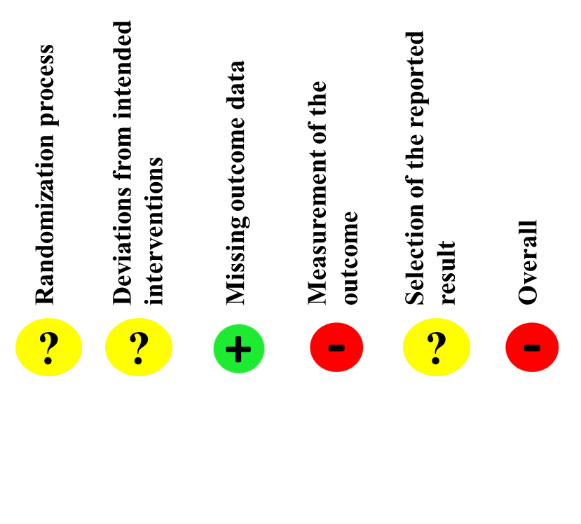 |
| Stephens et al., 1994 | RCT  (no blind assessors)  United States of America | **Criteria related to cannabis**  More than 50 times in the past 90 days  **Criteria related to other substances**  Excluded recent abuse or dependence on alcohol or  other drug | **Intervention group**  Relapse prevention group (80)  10 sessions  **Control group**  Social support group (87)  10 sessions | ***Severity of cannabis dependence***  Drug Abuse Screening Test  3-month: SMD=-0.12, CI=-0.43; 0.20  6-month: SMD=0.02, CI=-0.30; 0.33  12-month: SMD=0.08, CI=-0.23; 0.40  ***Frequency***  Self-developed questionnaire  1-month: SMD=-0.30, CI=-0.60; 0.01  3-month: SMD=-0.06, CI=-0.36; 0.24  6-month: SMD=-0.01, CI=-0.32; 0.29  9-month: SMD=0.12, CI=-0.18; 0.43  12-month: SMD=0.04, CI=-0.26; 0.34  ***Abstinence***  Self-developed questionnaire  3-month: OR=0.72, CI=0.38; 1.35  6-month: OR=0.81, CI=0.40; 1.64  12-month: OR=0.78, CI=0.35; 1.78 | 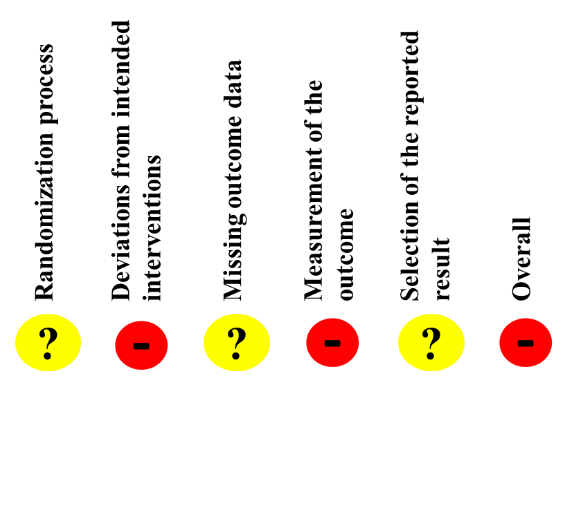 |
| **Authors, years** | **Design of studies**  **Studies countries** | **Description of sample** | **Comparisons groups** | **Results** | **Risk of bias** |
| Stephens et al., 2000 | RCT  (no blind assessors)  United States of America | **Criteria related to cannabis**  More than 50 times in the past 90 days  **Criteria related to other substances**  Excluded recent abuse or dependence on alcohol or other drug | **Intervention group**  Relapse prevention support group (CBT + social support processes) (103)  14 sessions  **Control group**  Brief MI (80)  2 sessions | ***Cannabis-related problems***  Adapted from other drug use severity instruments  Post: SMD=0.06, CI=-0.24; 0.36  3-month: SMD=0.06, CI=-0.25; 0.36  9-month: SMD=-0.10, CI=-0.40; 0.19  12-month: SMD=-0.10, CI=-0.39; 0.19  ***Severity of cannabis dependence***  Marijuana dependence scale  Post: SMD=0.01, CI=-0.30; 0.31  3-month: SMD=0.17, CI=-0.14; 0.47  9-month: SMD=0.00, CI=-0.29; 0.30  12-month: SMD=0.02, CI=-0.27; 0.32  ***Frequency***  Self-developed questionnaire  Post: SMD=-0.12, CI=-0.42; 0.19  3-month: SMD=0.04, CI=-0.26; 0.35  9-month: SMD=0.02, CI=-0.27; 0.32  12-month: SMD=-0.06, CI=-0.35; 0.23 | 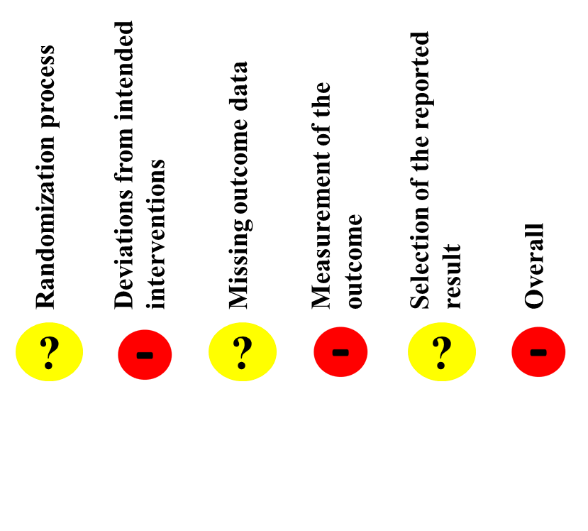 |
|  |  |  | **Intervention group**  Relapse prevention support group (CBT + social support processes) (95)  14 sessions  **Control group**  Delayed Treatment (79) | ***Cannabis-related problems***  Adapted from other drug use severity instruments  **Post: SMD=-1.03; CI=-1.35; -0.72**  ***Severity of cannabis dependence***  Marijuana dependence scale  **Post: SMD=-1.00, CI=-1.31; -0.68**  ***Frequency***  Self-developed questionnaire  **Post: SMD=-1.01; CI=-1.33; -0.69** |  |


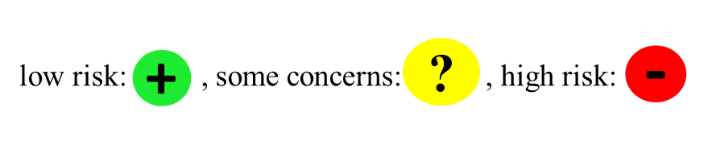

RT: randomized trial, RCT: randomized controlled trial, TAU: treatment as usual, MET: Motivational enhancement therapy, MI: Motivational interviewing, CM: Contingency management, CBT: cognitive behavioral therapy; TLFB: Timeline Follow-Back, NR: not report, SMD: standard mean difference, OR: Odd ratio, Significant results are in bold.

**Table S4.** PRISMA Checklist.

| **Section/topic** | **#** | **Checklist item** | **Reported on page #** |
| --- | --- | --- | --- |
| **TITLE** | | |  |
| Title | 1 | Identify the report as a systematic review, meta-analysis, or both. |  |
| **ABSTRACT** | | |  |
| Structured summary | 2 | Provide a structured summary including, as applicable, background, objectives, data sources, study eligibility criteria, participants, and interventions; study appraisal and synthesis methods; results; limitations; conclusions and implications of key findings; systematic review registration number. |  |
| **INTRODUCTION** | | |  |
| Rationale | 3 | Describe the rationale for the review in the context of what is already known. |  |
| Objectives | 4 | Provide an explicit statement of questions being addressed with reference to participants, interventions, comparisons, outcomes, and study design (PICOS). |  |
| **METHODS** | | |  |
| Protocol and registration | 5 | Indicate if a review protocol exists, if and where it can be accessed (e.g., Web address), and, if available, provide registration information including registration number. |  |
| Eligibility criteria | 6 | Specify study characteristics (e.g., PICOS, length of follow-up) and report characteristics (e.g., years considered, language, publication status) used as criteria for eligibility, giving rationale. |  |
| Information sources | 7 | Describe all information sources (e.g., databases with dates of coverage, contact with study authors to identify additional studies) in the search and date last searched. |  |
| Search | 8 | Present full electronic search strategy for at least one database, including any limits used, such that it could be repeated. |  |
| Study selection | 9 | State the process for selecting studies (i.e., screening, eligibility, included in systematic review, and, if applicable, included in the meta-analysis). |  |
| Data collection process | 10 | Describe method of data extraction from reports (e.g., piloted forms, independently, in duplicate) and any processes for obtaining and confirming data from investigators. |  |
| Data items | 11 | List and define all variables for which data were sought (e.g., PICOS, funding sources) and any assumptions and simplifications made. |  |
| Risk of bias in individual studies | 12 | Describe methods used for assessing risk of bias of individual studies (including specification of whether this was done at the study or outcome level), and how this information is to be used in any data synthesis. |  |
| Summary measures | 13 | State the principal summary measures (e.g., risk ratio, difference in means). |  |
| Synthesis of results | 14 | Describe the methods of handling data and combining results of studies, if done, including measures of consistency (e.g., I2) for each meta-analysis. |  |
| Risk of bias across studies | 15 | Specify any assessment of risk of bias that may affect the cumulative evidence (e.g., publication bias, selective reporting within studies). |  |
| Additional analyses | 16 | Describe methods of additional analyses (e.g., sensitivity or subgroup analyses, meta-regression), if done, indicating which were pre-specified. |  |
| **RESULTS** | | |  |
| Study selection | 17 | Give numbers of studies screened, assessed for eligibility, and included in the review, with reasons for exclusions at each stage, ideally with a flow diagram. |  |
| Study characteristics | 18 | For each study, present characteristics for which data were extracted (e.g., study size, PICOS, follow-up period) and provide the citations. |  |
| Risk of bias within studies | 19 | Present data on risk of bias of each study and, if available, any outcome level assessment (see item 12). |  |
| Results of individual studies | 20 | For all outcomes considered (benefits or harms), present, for each study: (a) simple summary data for each intervention group (b) effect estimates and confidence intervals, ideally with a forest plot. |  |
| Synthesis of results | 21 | Present results of each meta-analysis done, including confidence intervals and measures of consistency. |  |
| Risk of bias across studies | 22 | Present results of any assessment of risk of bias across studies (see Item 15). |  |
| Additional analysis | 23 | Give results of additional analyses, if done (e.g., sensitivity or subgroup analyses, meta-regression [see Item 16]). |  |
| **DISCUSSION** | | |  |
| Summary of evidence | 24 | Summarize the main findings including the strength of evidence for each main outcome; consider their relevance to key groups (e.g., healthcare providers, users, and policy makers). |  |
| Limitations | 25 | Discuss limitations at study and outcome level (e.g., risk of bias), and at review-level (e.g., incomplete retrieval of identified research, reporting bias). |  |
| Conclusions | 26 | Provide a general interpretation of the results in the context of other evidence, and implications for future research. |  |
| **FUNDING** | | |  |
| Funding | 27 | Describe sources of funding for the systematic review and other support (e.g., supply of data); role of funders for the systematic review. |  |
